# Molecular evolutionary rates predict both extinction and speciation in temperate angiosperm lineages

**DOI:** 10.1186/1471-2148-10-162

**Published:** 2010-06-01

**Authors:** Lesley T Lancaster

**Affiliations:** 1 National Center for Ecological Analysis and Synthesis. 735 State St., Suite 300. Santa Barbara, CA 93101, USA

## Abstract

**Background:**

A positive relationship between diversification (i.e., speciation) and nucleotide substitution rates is commonly reported for angiosperm clades. However, the underlying cause of this relationship is often unknown because multiple intrinsic and extrinsic factors can affect the relationship, and these have confounded previous attempts infer causation. Determining which factor drives this oft-reported correlation can lend insight into the macroevolutionary process.

**Results:**

Using a new database of 13 time-calibrated angiosperm phylogenies based on internal transcribed spacer (ITS) sequences, and controlling for extrinsic variables of life history and habitat, I evaluated several potential intrinsic causes of this correlation. Speciation rates (*λ*) and relative extinction rates (*ε*) were positively correlated with mean substitution rates, but were uncorrelated with substitution rate heterogeneity. It is unlikely that the positive diversification-substitution correlation is due to accelerated molecular evolution during speciation (e.g., via enhanced selection or drift), because punctuated increases in ITS rate (i.e., greater mean and variation in ITS rate for rapidly speciating clades) were not observed. Instead, fast molecular evolution likely increases speciation rate (via increased mutational variation as a substrate for selection and reproductive isolation) but also increases extinction (via mutational genetic load).

**Conclusions:**

In general, these results predict that clades with higher background substitution rates may undergo successful diversification under new conditions while clades with lower substitution rates may experience decreased extinction during environmental stasis.

## Background

Rates of molecular evolution often correlate positively with taxonomic diversity in angiosperms [1-6]. However, it has been difficult to distinguish among the many competing hypotheses for why clades with fast rates of nucleotide substitution (at various nuclear and organelle non-coding loci) also seem to be more speciose than clades with relatively conserved non-coding DNA regions. Hypothesized causes of the positive relationship between molecular evolution and diversification may be divided into two categories. Hypotheses for extrinsic causes suggest that aspects of a clade's ecology (its habitat or traits adapted to habitat) affect rates of both molecular evolution and diversification, but these rates *do not directly *affect each other (Hypothesis 1, Table 1). Hypotheses for intrinsic causes suggest that speciation or extinction events *directly *influence the average rate of molecular evolution for a clade, or conversely, suggest that rates of molecular evolution *directly *influence speciation or extinction events (Hypotheses 2 and 3, Table 1). A large body of previous work indicates that extrinsic, ecological effects are likely important contributors to the relationship between rates of diversification and molecular evolution. For example, shifts in environment, life history, or key innovations may subsequently influence both diversification and substitution rates [4,7,8]. However, it remains unclear whether there *also *remains a direct effect of diversification on rates of molecular evolution, or vice versa, when major ecological traits are accounted for. If such a direct relationship linking diversification and speciation rates does exist, then the form of this relationship may reveal evolutionary genetic processes involved in historical speciation events [9-11], potentially improving our understanding of when and how speciation and extinction will occur.

**Table 1:** Currently published hypotheses supported by a positive correlation between diversification and nucleotide substitution rates.

Extrinsic (Ecological) explanations:	1) Evolutionary rates are causally unrelated to the process of speciation, but are linked to traits or habitats that promote diversification.	1a) Life history. Increased generation time can cause mutations to accumulate faster over absolute time than they do in organisms with a slower life history [7,20,34] Shorter generation times are positively correlated with species richness of clades [8], possibly because shortened life cycles and/or enhanced mobility associated with smaller size allow individuals to colonize more extreme habitats.
		
		1b) Environmental energy. High levels of environmental energy such as UV radiation and temperature are mutagenic and tend to speed up development, resulting in increased rates of nucleotide substitution. Environmental energy is linked to both species richness and to rates of molecular evolution, but environmental energy does not appear to affect speciation rates via its effect on mutation rates. Instead, environmental energy directly affects both species-richness and faster evolution [4].
		
Intrinsic explanations:	2) Faster rates of molecular evolution in speciose clades are caused by rapid bursts of evolution during speciation events.	2a) Rapid evolution due to drift during speciation: If speciation commonly occurs in small, peripheral populations [e.g., founder event speciation, [10,35], then speciose clades will be more likely to have experienced reduced population sizes throughout their evolutionary history. Under conditions of reduced population sizes, selection is relaxed and slightly deleterious mutations may accumulate, contributing to enhanced substitution rates throughout the genome [9].
		
		2b) Rapid evolution due to increased selection pressure during speciation: Speciation involving adaptation to a new niche or geographic range will involve strong selection favoring traits adaptive in the new environment. In this process of adaptation, average population fitness may decline, reducing population sizes [19]. Selection is also more likely to favor mutations of large effect early in the process of adaptation [36]. The combined effects of smaller population sizes and selection for mutations of large effect might allow slightly deleterious and neutral mutations to hitchhike to fixation, causing rapid turnover of alleles throughout the genome during speciation.
		
	3) Faster rates of molecular evolution may cause speciation, by:	3a) Increasing the rate at which reproductive isolation develops between isolated populations [37].
		
		3b) Increasing the rate of production of new adaptations via increased mutational genetic variation [17].
		

Statistical artifact explanation:	4) Finally, the relationship between diversification and molecular evolution may be a statistical artifact.	When multiple substitutions have occurred at the same nucleotide position, this is undetectable on unbranched clades. However, if branching events have occurred between these 'multiple hits', then multiple substitutions at the same site are detectable. It will therefore appear as though the more speciose clade has undergone more substitutions (i.e., evolved faster) than its species-poor counterpart. This artifact is known as the node density effect [12,38].

Previous studies focused on establishing the ubiquity of the positive correlation between diversification and molecular evolution across plant clades, but often were unable to distinguish among the four major, published hypotheses for what drives the relationship (hypotheses listed in Table 1). These previous studies of evolutionary rates commonly employed sister group comparisons, obtaining the result that the more speciose sister clade has longer branches, on average, than its species-poor sibling. This measure is limited because it does not discriminate between the effects of speciation vs. extinction on extant diversity, leaving uncertainty in which of these two evolutionary processes are actually correlated with nucleotide substitution rates. Without controlling specifically for each of these factors, it is impossible to determine whether a direct relationship exists between the processes of either speciation or extinction and rates of nucleotide substitution. Furthermore, using sister group comparisons, it is impossible to directly test for variation in the tempo of evolution within clades (i.e., how 'clock-like' non-coding substitutions have been within that clade). If populational processes occurring during speciation directly cause the increases in average substitution rates observable across a phylogeny (Hypothesis 2 in Table 1, [6]), then substitution rate heterogeneity should also be observable across a phylogeny, with shorter branches (that have been in the process of speciation for a greater proportion of their span) exhibiting higher substitution rates than longer branches. However, previous studies have not tested for this. With sister-group comparisons, it is also difficult to rule out a confounding statistical artifact that affects the relationship between diversification and substitution rates, known as the node density effect (NDE, hypothesis 4 in Table 1). In sister-group comparisons, the response variable (species richness) is often correlated with the confounding variable (node density). Hugall and Lee [12] discuss methods to correct for this, and find that only by extensive resampling of the sister clades can one detect and account for NDE using sister group comparisons.

Instead of performing sister group comparisons, I constructed molecular-clock dated phylogenies for 13 angiosperm clades using internal transcribed spacer (ITS) regions of nuclear ribosomal DNA. Based on the generated phylogenies, I estimated speciation and extinction rates, the mean rate of nucleotide substitution, and the coefficient of variation in nucleotide substitution *within *each clade. These parameters were then compared *across *clades using phylogenetically independent contrasts (PIC's). By choosing 13 diverse angiosperm genera as independent clades for units of comparison rather than sister groups, I was able to determine if the correlation between diversification and substitution rates is robust and generalizable across a range of plants. With sister-groups, each clade can only directly be compared with its sibling. However, regression analysis of multiple, independent clades allows estimation of the general form of the relationship across angiosperms, which can be used to make general predictions.

I selected clades for comparison that exhibit similar life histories and environments, where ecological similarities across compared clades are not due to common ancestry (which could confound analyses). Although it is impossible to completely control for ecological differences, such an approach can reduce their magnitude of effect and allow me to determine if, after minimizing major differences in habit, geography, and life history, a relationship remains between substitution rates and speciation. If such a correlation exists, then I can tentatively rule out extrinsic, ecological explanations for variation in evolutionary rates (hypothesis 1 in Table 1) for the purposes of this study (i.e., reduce the magnitude of extrinsic effects on evolutionary rates from further analyses of intrinsic causes). If no relationship between speciation and substitution rates is detected after controlling for habitat and life history, I can reject an intrinsic, causal explanation (hypotheses 2 and 3).

Within each of the 13 clades under consideration, I calculated speciation and extinction using a birth-death diversification model, which calculates rates of speciation and extinction as independent parameters and allows for incomplete sampling of clades [13,14]. This method maximizes the likelihood of obtaining the observed tree, given particular values for speciation (*λ*) and extinction (*μ*) rates. Because *μ*depends on *λ*(i.e., extinction cannot occur without prior speciation), I calculated the relative extinction rate (ε = *λ*/*μ*) for use in further analyses. If substitution rates are positively correlated with speciation and uncorrelated with extinction, hypotheses 2 or 3a (Table 1) would be supported. If, instead, substitution rates are negatively correlated with extinction and uncorrelated with speciation, this would rule out both hypotheses 2 and 3 and would require a new, alternative hypothesis. If substitution rates correlate positively with both speciation and extinction, this would rule out hypothesis 2 and most strongly support hypothesis 3b. Maximum likelihood birth-death estimates of speciation and extinction rates, unlike other diversification measures, are not directly derived from the numbers of nodes over time, and should therefore be immune to NDE. These estimates can be compared to the effects of node density on substitution rates, thus directly controlling for hypothesis 4.

Using substitution rates derived from calibrated molecular clocks rather than from sister groups, I was also able to determine if the among-branch variation in the mean ITS substitution rate for each clade is correlated with increased speciation. If so, this would support hypothesis 2, suggesting that populational processes associated with speciation events cause accelerated bursts of molecular evolution and therefore increase the average rate of ITS substitution on short branches relative to the average rate for longer branches. If the regularity, or clock-like behavior, of ITS evolution is unaffected by the rate of speciation, this result would be more consistent with hypothesis 3.

## Results

**Figure 1:**
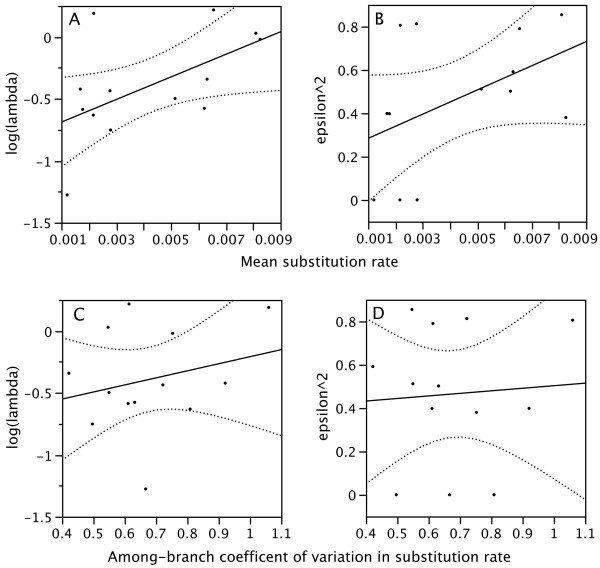
**Effects of the mean and among-branch variation in ITS substitution rate on speciation and extinction rates across temperate angiosperm clades**. a,b: The mean rate of ITS substitution predicts speciation (log[lambda]) and extinction (*ε*^2^), suggesting that elevated background mutation rates facilitate both of these processes. c,d: The coefficient of variation in substitution rate does not predict either speciation or extinction, suggesting that a punctuated evolution model is inappropriate to describe the relationship between rates of molecular evolution and speciation.

Values for *λ*, *ε*, and ITS substitution rates for each clade are listed in Table 2. Across clades, mean ITS substitution rate (the independent variable) was positively correlated with log(*λ*): Least squares regression of contrasts (through origin), r^2 ^= 0.298, slope = 105.02, F_1,11 _= 4.66, *P *= 0.05. Using branch lengths of 1, which corresponds to a punctuational (rather than gradual) model of trait evolution [15], least squares regression of contrasts (through origin), r^2 ^= 0.476, slope = 136.224, F_1,11 _= 9.975, *P *= 0.01. For the non-phylogenetic correlation (non-PIC), r^2 ^= 0.32, slope = 91.18, F_1,11 _= 5.16, *P *= 0.04 (Figure 1a). This positive relationship suggests that the correlation between diversification and rates of molecular evolution is due to a process of enhanced speciation rates in faster-evolving lineages, rather than decreased extinction. In further support of this conclusion, the mean ITS substitution rate was also marginally positively correlated with transformed values of relative extinction, ε^2^: Least squares regression of contrasts (through origin), r^2 ^= 0.191, slope = 58.30, F_1,11 _= 2.60, *P *= 0.13. For the PIC correlation assuming punctuated rate changes, r^2 ^= 0.276, slope = 64.28, F_1,11 _= 4.20, *P *= 0.06. For the non-PIC correlation, r^2 ^= 0.21, slope = 55.81, F_1,11 _= 2.95, *P *= 0.11 (Figure 1b). This marginally positive correlation suggests that faster substitution rates also promote or are otherwise associated with the process of extinction. A positive correlation between ITS rate and *ε *is inconsistent with hypothesis 2, and consistent with hypothesis 3.

**Table 2:** Evolutionary rates estimates.

Clade	*λ*	*95% C.I. (λ)*	μ	95% C.I. *(μ)*	*∑(μ/λ)*	mean ITS rate	95% HPD (mean rate)	ITS rate c.v.	95% HPD (c.v. rate)
Antirrhineae	1.070	(0.716, 1.588)	0.989	(0.602, 1.533)	0.924	8.10E-03	(6.24E-03, 1.02E-02)	0.547	(0.367, 0.737)
*Artemisia*	0.379	(0.275, 0.516)	0.239	(0.106, 0.401)	0.631	1.69E-03	(1.24E-03, 2.20E-03)	0.921	(0.697, 1.180)
Chironiinae	0.456	(0.292, 0.702)	0.350	(0.133, 0.637)	0.769	6.32E-03	(4.45E-03, 8.45E-03)	0.422	(0.245, 0.601)
*Ericameria*	0.234	(0.170, 0.342)	0	(0, 0.166)	0	2.17E-03	(1.01E-03, 3.82E-03)	0.809	(0.422, 1.214)
*Lepidium*	0.956	(0.670, 1.350)	0.589	(0.175, 1.086)	0.617	8.26E-03	(5.25E-03, 1.18E-02)	0.753	(0.512, 1.016)
*Lotus*	0.366	(0.259, 0.515)	0.330	(0.205, 0.492)	0.902	2.78E-03	(1.93E-03, 3.69E-03)	0.722	(0.514, 0.944)
*Lupinus*	1.547	(1.112, 2.139)	1.388	(0.900, 2.023)	0.898	2.18E-03	(1.26E-03, 3.28E-03)	1.060	(0.554, 1.631)
Lycieae	0.177	(0.136, 0.292)	0	(0, 0.163)	0	2.80E-03	(1.97E-03, 3.70E-03)	0.497	(0.246, 0.758)
Phrymoideae	0.265	(0.185, 0.375)	0.188	(0.079, 0.320)	0.709	6.22E-03	(4.79E-03, 7.72E-03)	0.632	(0.487, 0.790)
Polemoniaceae	0.318	(0.254, 0.396)	0.227	(0.145, 0.321)	0.715	5.15E-03	(4.25E-03, 6.11E-03)	0.551	(0.450, 0.656)
*Salvia*	1.649	(1.181, 2.282)	1.466	(0.957, 2.134)	0.889	6.56E-03	(5.13E-03, 8.04E-03)	0.614	(0.477, 0.760)
Saniculoideae	0.259	(0.194, 0.345)	0.164	(0.073, 0.269)	0.631	1.78E-03	(1.30E-03, 2.27E-03)	0.611	(0.442, 0.794)
*Sidalcea*, etc.	0.053	(0.039, 0.095)	0	(0, 0.061)	0	1.21E-03	(9.34E-04, 1.52E-03)	0.667	(0.419, 0.959)

Estimates for speciation rate (*λ*), extinction rate (*μ*), relative extinction rate (*∑*), mean rate of nucleotide substitution at the ITS1 and ITS2 loci (mean rate = number of substitutions per site/age in millions of years. It is calculated for each branch, then summed over all branches), and the coefficient of variation (c.v.) in mean substitution rate (c.v. rate = among-branch variance in ITS rate, scaled by the mean. I.e., for a tree with a c.v. of 0.50, the rate of ITS evolution varies across lineages by 50% of the mean). Confidence intervals (C.I.) for diversification rates were calculated from their likelihood profiles. 95% HPD (Highest Posterior Density) intervals for ITS rates are from BEAST (Tracer) analysis of MCMC output.

Variation among branches of the same tree in ITS sequence evolution (coefficient of variation in mean rate) was not correlated with log(*λ*) (PIC: *P *= 0.23, non-PIC: *P *= 0.43, Figure 1c) nor with *ε*^2 ^(PIC: *P *= 0.62, non-PIC: *P *= 0.83, Figure 1d), suggesting that neither increased speciation nor extinction events introduced increased deviations from the background pace of non-coding molecular evolution. This result (of no relationship between speciation rate and a punctuated nature of nucleotide evolution), in addition to the suggested positive relationship between substitution rates and extinction, refutes hypothesis 2.

In contrast to the positive relationship between ITS substitution rates and log(*λ*), substitution rates were uncorrelated with node density (non-PIC: *P *= 0.39). This lack of correlation confirms that the positive relationship between maximum likelihood speciation and extinction parameters and substitution rates calculated by BEAST are not artifacts of NDE, ruling out hypothesis 4. Furthermore, the number of sequences used in constructing each tree did not affect the mean substitution rate (*P *= 0.74) or the c.v. in substitution rate (*P *= 0.92), ruling out the possibility that the positive relationship between log(*λ*) and mean ITS rate was spuriously generated by sampling more sequences in some clades, which could increase the chances of sampling a lineage with a high substitution rate by chance. Finally, clade age was uncorrelated with mean substitution rate (*P *= 0.42), indicating that the significant correlations reported here were not spuriously caused by calibration date uncertainty.

## Discussion

My choice of clades was designed to minimize effects of the most-commonly reported potential ecological drivers of this relationship, habit and biogeographical range. After partially controlling for these predominating ecological drivers of diversification and mutation rate, a relationship exists between ITS substitution rates and both speciation and, marginally, extinction. These results suggest that a direct, potentially causal, intrinsic relationship exists between the average rate of molecular evolution within a clade, and the rate of both speciation and extinction within that clade.

The hypothesis best supported by these results is 3b, indicating that clades with higher rates molecular evolution at non-coding loci (such as ITS) likely also experience higher rates of molecular evolution at coding loci (see also [5]). An increase in mutational genetic variation causes increases in phenotypic variation that can facilitate both speciation (via increased adaptive potential in new niches or marginal environments) and extinction (via increased mutational load [16,17]). These results do not rule out hypothesis 3a, however. Although hypothesis 3a does not explain the marginally significant relationship between substitution and extinction rates (which best supports hypothesis 3b), it is also possible that a fast rate of molecular evolution increases the rate at which genetic incompatibilities accumulate between populations. Further studies incorporating information on mating system and the hybridization abilities within these clades are needed to determine if this is the case.

These results provide evidence to falsify hypothesis 2, suggesting that rates of molecular evolution may not provide a 'signature' of past speciation events [9]. Therefore, analysis of rate heterogeneity may not allow us to distinguish historical processes of speciation (i.e., vicariant vs. peripatric speciation). When testing specifically for a relationship between speciation rates and the coefficient of variation for substitution rates (high variation indicates punctuated changes in substitution rates), the relationship was found to be absent in this data set. This indicates that while substitution rates might briefly increase during speciation events, these interludes cannot be detected in the phylogeny using current methods; overall higher rates of substitution observed across the tree for speciose clades are not caused by punctuated increases at speciation. These results suggest that the result of faster evolution in more speciose clades is not sufficient evidence to support the punctuated evolution model of speciation (as claimed by [1,6]).

Because these results suggest that the rate of nucleotide substitution is a cause rather than an effect of speciation, this leaves the causes of variation in rates of substitution to be explained. Substitution rates are in part determined by the efficiency of DNA replication and repair mechanisms [18]. Potential explanations for the differences in substitution rates among clades are that substitution rates are phylogenetically conserved, or that they are correlated with genome size (larger genomes may contain more 'junk DNA' and thus experience either relatively lower genome-wide selection against increased mutation rates or higher time and energy costs of more accurate replication). However, using this data set, I did not detect a phylogenetic signal for substitution rate (see also [7]) or any correlation of substitution rate with average genome size within each clade (Lancaster, unpublished data). Finally, finer distinctions between the ecologies of these clades than what I was able to control for here likely also affect the reported relationships. In future work with these clades, I will examine fine-scale ecological effects on diversification.

## Conclusions

Plant species from clades characterized by high nucleotide substitution rates tend to both speciate and go extinct at higher rates than species from more slowly-evolving clades. High substitution rates are due to large mutational genetic variance experienced at the population level, which increases both adaptive potential within populations and reproductive isolation between populations, which can facilitate the process of speciation. However, elevated mutational genetic variation also leads to extinction, likely via the genetic load it imposes. The causes of substitution rate variation across clades with similar ecologies are not well known.

One important question generated by these results is whether substitution rates can be applied to predict these and other clades' responses to environmental change. The ability of a population to adapt to a rapidly changing environment is directly proportional to its mutation rate [19], which provides the necessary phenotypic variation to allow populations to respond to novel selection pressures. The results reported here suggest that high mutation rates also allow clades to speciate more rapidly, diversifying into new niches (potentially as new niches arise). Clades exhibiting fast substitution rates may therefore be more likely survive rapidly changing or novel environments, in spite of the fact that they otherwise have increased chances of extinction because of their relatively higher levels of mutational genetic load.

## Methods

### Clade selection

For phylogenetic analyses, I selected thirteen temperate angiosperm genera or tribes that each contained enough species (a large enough within-clade sample size) to accurately calculate diversification rates, with mean ITS-sampled clade size = 117.08, minimum = 47, maximum = 304. I selected clades that were relatively completely sampled for ITS sequence data, and for which fossil or vicariance data, or previously published ITS rate data, was available to calibrate molecular clocks. For the 11 out of 13 clades in which fossil or vicariance dates were used to calibrate trees, 2-3 dating events per tree were used to increase accuracy (Table 3). For the remaining 2 clades without known fossils, well-established calibration points from the literature were available, based on previous molecular clock analyses of more inclusive clades (references in Table 3). Although variation in the quality of information available to calibrate trees likely varies from clade to clade, this potential source of noise in the data is not expected to introduce systematic bias [7]. To attempt to control for life history, I chose clades exhibiting similar growth habits, because growth habit is strongly correlated with generation time and reproductive strategy, and is commonly used as a proxy for life history in phylogenetic studies [7,20]. The 13 clades that I chose each consisted predominantly of a mixture of perennial herbs and low shrubs [plant height may affect substitution rate, [21]]. Clades containing shrubs tend to fossilize well compared to herbaceous clades, and clades containing herbs provide larger clade sizes, with increased power to estimate diversification rates, than do genera or tribes composed entirely of woody species. The 13 clades varied in their proportions of woody vs. herbaceous members, however, which could affect the results. To control for geography, I selected clades that had diversified within and occupy overlapping ranges in temperate North America, and that have a predominantly temperate distribution in the remainder of their ranges. Of course I could not find sufficient numbers of replicate clades with completely overlapping ranges, so geographical variation could also affect these results. Latitude and tropical vs. temperate ranges are the most commonly reported habitat variables affecting diversification and rates of substitution [4,22]. Although both of these variables are imperfectly matched across clades, I worked to minimize their influence without compromising my criteria of random clade selection, extensive ITS sampling, and reliable calibration dates. The thirteen clades finally selected as fulfilling all of these criteria are as follows: The tribe Antirrhineae (Plantaginaceae), *Artemisia *(Asteraceae), the tribe Chironiinae (Gentianaceae), *Ericameria *(Asteraceae), *Lepidium *(Brassicaceae), *Lotus *(Fabaceae), *Lupinus *(Fabaceae), the tribe Lycieae (Solanaceae), the subfamily Phrymoideae (Phrymaceae), the family Polemoniaceae, *Salvia *(Lamiaceae), the subfamily Saniculoideae (Apiaceae), and *Sidalcea *plus *Calyculogygas, Eremalche*, *Iliamna, Malvastrum, Modiola, Modiolastrum*, and *Monteiroa *[monophyly of these genera from [23]]; Malvaceae). I also selected an appropriate outgroup for each clade, which was not included in diversification analyses.

**Table 3:** Nucleotide substitution models and calibration dates for each of the 13 clades under consideration.

Clade	Substitution model	Calibration Priors				
		Node	age (mya)	source	citation	distribution *
Antirrhineae	GTR+I+Γ	Ingroup	38-48	previous estimate	[39]	Normal, 43, 3
		*Acanthorrhinum, Albraunia, Antirrhinum, Chaenorhhinum, Galvezia, Holzneria, Misopates, Mohavea*, and *Pseudomisopates*	21.49 ± 4.27	previous estimate	[39]	Normal, 21.49, 6

*Artemisia*	GTR+Γ	Ingroup	34 - 45	fossil and previous estimate	[40-42]	Uniform, 34, 45
		*A. canariensis - A. arborescens *split	17.1	vicariance event	[43]	Uniform, 0, 17.1

Chironiinae	GTR+Γ	Ingroup	54.8	fossil - earliest Gentianaceae	[44]	Uniform, 0, 54.8
		Mexican species of *Zeltnera*	5	vicariance event	[45]	Normal, 5, 0.2
		*Z. trichantha - Z. namophila *split	2	vicariance event	[45]	Normal, 2, 0.2

*Ericameria *	GTR+Γ	Ingroup	5-26	previous estimate	[46]	Uniform, 5, 26
		*E. ophitidis - E. gilmanii *split	5	vicariance event	[47]	Normal, 5, 1.0

*Lepidium*	GTR+Γ			ITS rate for Brassicaceae	[48]	Uniform, 4.5E-3, 8.3E-3 subst/site/my

*Lotus*	GTR+Γ	Ingroup	65	fossil	[49]	Lognormal, 65, 0, 1
		Canary Island clade	0-21	vicariance event	[50]	Normal, 12, 3

*Lupinus*	GTR+I+Γ	Ingroup	18	previous estimate	[51]	Normal, 18, 2.5

Lycieae	GTR+I+Γ	Ingroup	86-20	previous estimate	[52,53]	Uniform, 20, 86
		*Lycium*	29.4 ± 9.7	previous estimate	[52]	Normal, 29.5, 5.9
		Australian-Eurasian split	14.1 ± 3.2	previous estimate	[52]	Normal, 14.1, 1.945

Phrymoideae	GTR+I+Γ	Ingroup + *Verbina*	49.4	previous estimate - minimum	[54]	Lognormal, 49.4, 0, 1
		*Phryma*	3.93	previous estimate	[54]	Normal, 3.93, 2.46

Polemoniaceae	GTR+I+Γ	Ingroup	41.3 - 91.2	previous estimate	[55,56]	Normal, 50, 5
		Giliae	41.3	fossil	[55]	Lognormal, 41.3, 0, 1

*Salvia *	GTR+I+Γ	Ingroup	25	fossil	[57,58]	Lognormal, 25, 0, 1
		*S. glutinosa - *(*S. digitaloidies, S. robrowskii, S. cynica, S. przewalskii*) *split*	11.61 - 3.6	fossil	[59]	Lognormal, 3.6, 2, 1
		*S. officinalis - S. aucheri *split	3.6 - 2.59	fossil	[60]	Lognormal, 2.69, 2, 1

Saniculoideae	GTR+Γ	Ingroup	33.7 - 69	fossil and previous estimate	[53,61,62]	Lognormal, 33.7, 2, 1
		*Sanicula*	33.7	fossil	[61]	Lognormal, 33.7, 0, 1

*Sidalcea*, etc.	GTR+Γ	Ingroup	34 - 69.7	fossil and previous estimate	[56,63]	Uniform, 34, 70
		*Malvastrum*	34	fossil	[63]	Lognormal, 0, 1

*Values separated by commas represent the following parameters: Normal, mean, standard deviation (s.d.); Uniform, lower bound, upper bound; or Lognormal, zero offset, mean, s.d.

Units are in m.y. (millions of years) unless otherwise indicated.

### Phylogeny and Molecular Clock analyses

I obtained sequences for the ITS-1 and ITS-2 regions of 18s-26s nuclear ribosomal DNA from Genbank (http://www.ncbi.nlm.nih.gov; accession numbers provided in Additional file 1). ITS sequences have been found highly useful in phylogenetic studies because they are readily obtained in the laboratory, and provide high phylogenetic resolution at the species level [24]. Therefore, ITS sequences are available for many species, facilitating large-scale comparisons such as in this study. I aligned the ITS sequences within each clade in MUSCLE v3.7 [25], using default parameters. Poorly-aligning regions and coding regions of 18s, 5.8s, or 26s nrDNA were assessed by eye in Mesquite v2.6 and 2.7 [26] and were clipped from the final alignments. For each clade, I imported aligned sequences into PAUP* v4.0 [27] in order to run MrModelTest v2.3 [28], to determine the appropriate model of nucleotide substitution according the hLRT criteria. Selected substitution models for each clade are presented in Table 3.

I constructed time-calibrated phylogenies using a Bayesian Markov Chain Monte-Carlo (MCMC) method implemented in BEAST v4.8 and 5.0 [29] using a lognormally distributed relaxed molecular clock model [30] and a birth-death tree prior. I initially evaluated phylogenies with an MCMC chain length of 10,000,000 states, but increased chain length to as high as 50,000,000 for clades with low ESS (effective sample sizes) of resulting tree parameters. Priors for dating events used to calibrate each molecular clock are reported in Table 3. Multiple BEAST runs were compared and combined to generate a final tree for each clade, after removing a burn-in of 10% of the chain length for each BEAST run. The resulting mean rate parameter (which is the average of the individual substitution rates along each branch, weighted by branch length, to provide a measure of the overall substitution rate per site per million years) and the coefficient of variation in the mean rate parameter (a measure of how clock-like ITS evolution has been) are listed for each clade, with further explanation, in Table 2.

### Diversification rate analysis and independent contrast analysis of tree parameters

Lambda (*λ*, speciation rate) and mu (*μ*, extinction rate) were calculated for each of the calibrated trees in the DiversiTree v.0.4-1 module [13] of R v.2.9.2 http://www.R-project.org, using a standard birth-death diversification model [14]. Because my selected clades were incompletely sampled, I applied the correction built into the DiversiTree module to account for the effect of sampling frequency on estimated rates. Confidence intervals for these rates were derived from the likelihood profile. Because *λ*and *μ*are positively correlated (extinction rates depend on prior speciation events), I calculated the relative extinction rates (*ε*) as *λ*/*μ*for further analysis of the relationship between substitution and extinction rates. The 13 values of *λ*and *ε *that I obtained (Table 2) were not normally distributed, and I therefore log-transformed *λ*and squared the values of *ε *to achieve normality.

I evaluated relationships between diversification measures (log(*λ*) and *ε*^2^) and substitution rates both directly in Jump v7.0 (SAS institute, ^©^2007) and using phylogenetically independent contrasts (PIC's) implemented in the PDAP package of Mesquite [15,31]. PIC's transform character states at tips to incorporate information about branching. This method corrects for any potential sources of pseudoreplication due to shared ancestry of related clades, allowing clades to be treated as statistically independent subjects. To calculate PIC's, I used a phylogeny including all thirteen clades (i.e., each of my 13 selected clades resides at a tip) retrieved from Phylomatic's maximally resolved seed plant tree [32], with branch lengths proportional to time (divergence dates from [33]). The PDAP diagnostic chart was used to determine that these branch lengths were acceptable. Using this tree for branching information between my selected clades, I evaluated the correlation among clades between mean substitution rate and the diversification rates log(*λ*) and *ε*^2^. I also evaluated the correlation between the coefficient of variation in substitution rate and log(*λ*) and *ε*^2^, to test whether bursts in substitution rates accompanying speciation were evidenced by less clock-like ITS evolution in more speciose clades (hypothesis 2). To test if the substitution rate calculated in BEAST depends on node density (which could cause spurious results), I verified that mean substitution rate was uncorrelated with node density (number of nodes/clade age). I also verified that mean ITS rate was uncorrelated with clade age. This was done because uncertainty in dating phylogenies could lead to over- or under-estimation of some clade ages, generating spurious correlations between evolutionary rates. A correlation between clade age and substitution rate would indicate spurious rate correlations caused by calibration errors.

## Supplementary Material

Additional file 1 **Genbank accession numbers for internal transcribed spacer sequences used in phylogenetic tree construction**. Click here for file
