# Supplementary material for: Molecular evolutionary rates predict both extinction and speciation in temperate angiosperm lineages
Source: BMC Evol Biol. 2010 Jun 1;10:162. doi: 10.1186/1471-2148-10-162 (PMC2901258; doi:10.1186/1471-2148-10-162)
Supplement: Additional file 1 — Genbank accession numbers for internal transcribed spacer sequences used in phylogenetic tree construction. [file 1471-2148-10-162-S1.DOC]

|  |  | ITS 1 | ITS 2 | ITS 1 and 2 |
| --- | --- | --- | --- | --- |
| Antirrhineae |  |  |  |  |
|  | *Acanthorrhinum ramosissimum* |  |  | AY731261 |
|  | *Albraunia foveopilosa* |  |  | AY731250 |
|  | *Anarrhinum bellidifolium* |  |  | AY878116 |
|  | *Anarrhinum fruticosum* |  |  | AF513881 |
|  | *Antirrhinum australe* |  |  | EU677195 |
|  | *Antirrhinum barrelieri* |  |  | AY880240 |
|  | *Antirrhinum braun-blanquetii* |  |  | EU677197 |
|  | *Antirrhinum breweri* |  |  | AY880229 |
|  | *Antirrhinum charidemi* |  |  | FJ487611 |
|  | *Antirrhinum controversum* |  |  | EU677207 |
|  | *Antirrhinum cornutum* |  |  | AF513905 |
|  | *Antirrhinum costatum* |  |  | AF513893 |
|  | *Antirrhinum coulterianum* |  |  | AF513890 |
|  | *Antirrhinum cyathiferum* |  |  | AF513884 |
|  | *Antirrhinum filipes* |  |  | AF513896 |
|  | *Antirrhinum graniticum* |  |  | EU677209 |
|  | *Antirrhinum grosii* |  |  | EU677210 |
|  | *Antirrhinum hispanicum* |  |  | FJ487614 |
|  | *Antirrhinum kelloggii* |  |  | AF513904 |
|  | *Antirrhinum kingii* |  |  | AF513903 |
|  | *Antirrhinum latifolium* |  |  | EU677213 |
|  | *Antirrhinum leptaleum* |  |  | AF513906 |
|  | *Antirrhinum linkianum* |  |  | AY731278 |
|  | *Antirrhinum litigiosum* |  |  | AY731277 |
|  | *Antirrhinum lopesianum* |  |  | EU677218 |
|  | *Antirrhinum majus* |  |  | FJ648325 |
|  | *Antirrhinum meonanthum* |  |  | EU677220 |
|  | *Antirrhinum microphyllum* |  |  | EU677223 |
|  | *Antirrhinum molle* |  |  | FJ487612 |
|  | *Antirrhinum mollissimum* |  |  | AF513886 |
|  | *Antirrhinum multiflorum* |  |  | AF513897 |
|  | *Antirrhinum nuttallianum* |  |  | AF513895 |
|  | *Antirrhinum orontium* |  |  | AF513889 |
|  | *Antirrhinum ovatum* |  |  | AF513899 |
|  | *Antirrhinum pertegasii* |  |  | EU677228 |
|  | *Antirrhinum pulverulentum* |  |  | EU677234 |
|  | *Antirrhinum sempervirens* |  |  | EU677236 |
|  | *Antirrhinum siculum* |  |  | FJ648327 |
|  | *Antirrhinum subbaeticum* |  |  | EU677241 |
|  | *Antirrhinum subcordatum* |  |  | AF513902 |
|  | *Antirrhinum tortuosum* |  |  | AY731285 |
|  | *Antirrhinum valentinum* |  |  | EU677246 |
|  | *Antirrhinum vexillo-calyculatum* |  |  | AF513901 |
|  | *Antirrhinum virga* |  |  | AF513898 |
|  | *Antirrhinum watsonii* |  |  | AF513894 |
|  | *Asarina procumbens* |  |  | AY880236 |
|  | *Chaenorrhinum minus* |  |  | AF513875 |
|  | *Chaenorrhinum tenellum* |  |  | AY731251 |
|  | *Cymbalaria muralis* |  |  | AF513883 |
|  | *Galvezia fruticosa* |  |  | AF513885 |
|  | *Galvezia juncea* |  |  | AY880243 |
|  | *Galvezia limensis* |  |  | AY492104 |
|  | *Galvezia speciosa* |  |  | AY316309 |
|  | *Holzneria spicata* |  |  | AY731258 |
|  | *Kicksia elatine* |  |  | AY880225 |
|  | *Kicksia spuria* |  |  | AF513880 |
|  | *Linaria genistifolia* |  |  | AY878115 |
|  | *Linaria hirta* |  |  | AY731245 |
|  | *Linaria micrantha* |  |  | AY731242 |
|  | *Linaria repens* |  |  | AY731246 |
|  | *Linaria spartea* |  |  | AY731247 |
|  | *Linaria triornithophora* |  |  | AY731248 |
|  | *Linaria vulgaris* |  |  | DQ531053 |
|  | *Lophospermum erubescens* |  |  | AY731249 |
|  | *Maurandya antirrhiniflora* |  |  | AF513878 |
|  | *Maurandya wislizeni* |  |  | AY878930 |
|  | *Misopates calycinum* |  |  | AY731259 |
|  | *Mohavea breviflora* |  |  | AF513892 |
|  | *Mohavea confertiflora* |  |  | AF513891 |
|  | *Pseudomisopates rivas-martinezii* |  |  | FJ897735 |
|  | *Rhodochiton* sp. |  |  | AF513876 |
|  | *Schweinfurthia imbricata* |  |  | AY731254 |
|  | *Schweinfurthia latifolia* |  |  | AY731255 |
|  | *Schweinfurthia papilionacea* |  |  | AY731253 |
|  | *Schweinfurthia pedicellata* |  |  | AY731256 |
|  | *Schweinfurthia pterosperma* |  |  | AF513882 |
|  | *Schweinfurthia spinosa* |  |  | AY731257 |
|  | Outgroup: *Plantago lanceolata* |  |  | EU036287 |
| *Artemisia* |  |  |  |  |
|  | *Artemisia abrotanum* | AF060473 | AF061390 |  |
|  | *Artemisia absinthum* | AY180178 | AY180179 |  |
|  | *Artemisia adamsii* |  |  | AM398844 |
|  | *Artemisia afra* | AF045392 | AF140484 |  |
|  | *Artemisia agrillosa* | EU111676 | EU111677 |  |
|  | *Artemisia alaskana* |  |  | AM398845 |
|  | *Artemisia androsacea* |  |  | AM398846 |
|  | *Artemisia annua* | AY131969 | AF079935 |  |
|  | *Artemisia apiacea* |  |  | AM398848 |
|  | *Artemisia araxina* | AF045408 | AF079959 |  |
|  | *Artemisia arborescens* | AY180180 | AY180181 |  |
|  | *Artemisia arbuscula* | AF060464 | AF061380 |  |
|  | *Artemisia arctica* |  |  | AM398849 |
|  | *Artemisia arenaria* | EF063639 | EF063640 |  |
|  | *Artemisia argyi* | DQ925699 | DQ925700 |  |
|  | *Artemisia aschurbajewii* | AF504170 | AF504143 |  |
|  | *Artemisia austriaca* | AF504171 | AF504144 |  |
|  | *Artemisia bargusinensis* |  |  | AM398850 |
|  | *Artemisia barrelieri* | AF045410 | AF079961 |  |
|  | *Artemisia biennis* | AY131967 | AY131968 |  |
|  | *Artemisia bigelovii* | AF060464 | AF061385 |  |
|  | *Artemisia borealis* |  |  | AM398852 |
|  | *Artemisia caerulescens* | AF045409 | AF079960 |  |
|  | *Artemisia caespitosa* |  |  | AM398855 |
|  | *Artemisia californica* | AF060474 | AF061388 |  |
|  | *Artemisia campestris* | AF045398 | AF079950 |  |
|  | *Artemisia cana* | AF045413 | AF079965 |  |
|  | *Artemisia canadensis* |  |  | AM398856 |
|  | *Artemisia canariensis* | DQ028920 | DQ028907 |  |
|  | *Artemisia capillaris* |  |  | AY548201 |
|  | *Artemisia chamaemelifolia* | AF045388 | AF079940 |  |
|  | *Artemisia changaica* |  |  | AM398858 |
|  | *Artemisia comata* |  |  | AM398859 |
|  | *Artemisia commutata* |  |  | AM398860 |
|  | *Artemisia compacta* |  |  | AM398861 |
|  | *Artemisia crithmifolia* | AF045399 | AF079962 |  |
|  | *Artemisia czekanowskiana* |  |  | AM398862 |
|  | *Artemisia depauperata* |  |  | AM398863 |
|  | *Artemisia diversifolia* | AF504192 | AF504165 |  |
|  | *Artemisia dolosa* |  |  | AM398864 |
|  | *Artemisia dracunculiformis* |  |  | AM398865 |
|  | *Artemisia dracunculoides* | AF504172 | AF504145 |  |
|  | *Artemisia dracunculus* | AF045401 | AF079952 |  |
|  | *Artemisia eranthema* | AF504195 | AF504168 |  |
|  | *Artemisia eriantha* | DQ028919 | DQ028906 |  |
|  | *Artemisia eriocarpa* | AF504191 | AF504164 |  |
|  | *Artemisia filifolia* | DQ028922 | DQ028909 |  |
|  | *Artemisia flava* |  |  | AM398867 |
|  | *Artemisia fragrans* | AF045406 | AF079957 |  |
|  | *Artemisia freyniana* |  |  | AM398868 |
|  | *Artemisia frigida* |  |  | AM398869 |
|  | *Artemisia furcata* |  |  | AM398870 |
|  | *Artemisia genipi* | AY180186 (mislabeled its2) | AY180187 |  |
|  | *Artemisia glacialis* | DQ028921 | DQ028908 |  |
|  | *Artemisia glauca* |  |  | AM398871 |
|  | *Artemisia globularia* |  |  | AM398872 |
|  | *Artemisia glomerata* |  |  | AM398873 |
|  | *Artemisia gmelinii* |  |  | AM398875 |
|  | *Artemisia gobica* |  |  | AM398876 |
|  | *Artemisia gorgonum* |  |  | AM398877 |
|  | *Artemisia granatensis* | AF045397 | AF079949 |  |
|  | *Artemisia haussknechtii* | AF504173 | AF504146 |  |
|  | *Artemisia herba-alba* | AF045403 | AF079955 |  |
|  | *Artemisia hultenii* |  |  | AM398878 |
|  | *Artemisia hyperborea* |  |  | M398879 |
|  | *Artemisia incanescens* | AF504174 | AF504147 |  |
|  | *Artemisia inculta* | AF045405 | AF079956 |  |
|  | *Artemisia indica* |  |  | EF107651 |
|  | *Artemisia integrifolia* |  |  | AM398880 |
|  | *Artemisia iwayomogi* |  |  | AY548202 |
|  | *Artemisia jacutica* |  |  | AM398881 |
|  | *Artemisia japonica* |  |  | AM398882 |
|  | *Artemisia judaica* | AF504175 | AF504148 |  |
|  | *Artemisia klementzae* |  |  | AM398883 |
|  | *Artemisia koidzumii* |  |  | AM398884 |
|  | *Artemisia kruhsiana* |  |  | AM398885 |
|  | *Artemisia laciniata* |  |  | AM398886 |
|  | *Artemisia laciniatiformis* |  |  | AM398887 |
|  | *Artemisia lagocephala* |  |  | AM398888 |
|  | *Artemisia lagopus* |  |  | AM398889 |
|  | *Artemisia leucodes* | AF504176 | AF504149 |  |
|  | *Artemisia leucophylla* |  |  | AM398890 |
|  | *Artemisia longiloba* | AF060467 | AF061381 |  |
|  | *Artemisia lucentica* | AF045390 | AF079943 |  |
|  | *Artemisia ludoviciana* | EU124795 | EU124798 |  |
|  | *Artemisia macrantha* |  |  | AM398892 |
|  | *Artemisia manshurica* |  |  | AM398893 |
|  | *Artemisia marschalliana* | AF504177 | AF504150 |  |
|  | *Artemisia messerschmidtiana* |  |  | AM398894 |
|  | *Artemisia mexicana* | AF045414 | AF079966 |  |
|  | *Artemisia michauxiana* |  |  | AM398895 |
|  | *Artemisia molinieri* | AF045389 | AF079941 |  |
|  | *Artemisia monosperma* | AF045400 | AF079951 |  |
|  | *Artemisia monostachya* |  |  | AM398896 |
|  | *Artemisia nesiotica* | EU111674 | EU111675 |  |
|  | *Artemisia norvegica* |  |  | AM398897 |
|  | *Artemisia nova* | AF045412 | AF079964 |  |
|  | *Artemisia obscura* |  |  | AM398899 |
|  | *Artemisia obtusiloba* |  |  | AM398900 |
|  | *Artemisia opulenta* |  |  | AM398901 |
|  | *Artemisia palmeri* | AF504178 | AF504151 |  |
|  | *Artemisia palustris* |  |  | AM398902 |
|  | *Artemisia pamirica* |  |  | AM398903 |
|  | *Artemisia papposa* | EU111668 | EU111669 |  |
|  | *Artemisia parviflora* | AB359697 | AB359783 |  |
|  | *Artemisia pedatifida* | EU111672 | EU111673 |  |
|  | *Artemisia pedemontana* | AY180192 | AY180193 |  |
|  | *Artemisia persica* | AF504179 | AF504152 |  |
|  | *Artemisia phaeolepis* |  |  | AM398905 |
|  | *Artemisia princeps* | AY180194 | AY180195 |  |
|  | *Artemisia pubescens* |  |  | AM398907 |
|  | *Artemisia punctigera* |  |  | AM398908 |
|  | *Artemisia pycnorhiza* |  |  | AM398909 |
|  | *Artemisia pygmaea* | AF060468 | AF061384 |  |
|  | *Artemisia reptans* | AF045391 | AF079944 |  |
|  | *Artemisia rigida* | AF060465 | AF061382 |  |
|  | *Artemisia rothrockii* | EU124794 | EU124797 |  |
|  | *Artemisia rupestris* | AF060475 | AF061391 |  |
|  | *Artemisia rutifolia* | AF504180 | AF504153 |  |
|  | *Artemisia salsoloides* | AB359699 | AB359785 |  |
|  | *Artemisia samoiedorum* |  |  | AM398912 |
|  | *Artemisia santolina* | AF504181 | AF504154 |  |
|  | *Artemisia santolinifolia* | AF504182 | AF504155 |  |
|  | *Artemisia scoparia* | AF045402 | AF079953 |  |
|  | *Artemisia senjavinensis* |  |  | AM398915 |
|  | *Artemisia sericea* |  |  | EF577290 |
|  | *Artemisia serrata* | AF514345 | AF514346 |  |
|  | *Artemisia sibirica* | AF504187 | AF504160 |  |
|  | *Artemisia sieberi* | AF045407 | AF079958 |  |
|  | *Artemisia sieversiana* | AF504183 | AF504156 |  |
|  | *Artemisia sphaerocephala* |  |  | AM398918 |
|  | *Artemisia spiciformis* | EU111666 | EU111667 |  |
|  | *Artemisia spinescens* | AF504184 | AF504157 |  |
|  | *Artemisia splendens* | AF045396 | AF079948 |  |
|  | *Artemisia stelleriana* |  |  | AM398919 |
|  | *Artemisia subarctica* |  |  | AM398920 |
|  | *Artemisia sublessingiana* | AF060478 | AF061394 |  |
|  | *Artemisia subviscosa* |  |  | AM398921 |
|  | *Artemisia suksdorfii* | AF514347 | AF514348 |  |
|  | *Artemisia superba* |  |  | AM398922 |
|  | *Artemisia tanacetifolia* |  |  | AM398923 |
|  | *Artemisia thuscula* |  |  | AM398924 |
|  | *Artemisia tilesii* | AF514353 | AF514354 |  |
|  | *Artemisia tournefortiana* | AF045384 | AF079936 |  |
|  | *Artemisia tridentata* | AF045411 | AF079963 |  |
|  | *Artemisia tripartita* | AF060463 | AF061379 |  |
|  | *Artemisia umbelliformis* | AY180196 | AY180197 |  |
|  | *Artemisia unalaskensis* |  |  | AM398926 |
|  | *Artemisia verlotiorum* | AF045387 | AF079939 |  |
|  | *Artemisia vulgaris* | AY180198 | AF514352 |  |
|  | *Artemisia xanthochroa* |  |  | AM398928 |
|  | *Artemisia xerophytica* |  |  | AM398929 |
|  | Outgroup: *Kaschgaria komarovii* | AY127689 | AY127690 |  |
| Chironiinae |  |  |  |  |
|  | *Blackstonia acuminata* | AY251685 | AY251715 |  |
|  | *Blackstonia grandiflora* | AY251684 | AY251714 |  |
|  | *Blackstonia imperfoliata* | AJ011470 | AJ011480 |  |
|  | *Blackstonia perfoliata* | AY047793 | AY047878 |  |
|  | *Centaurium barrelieri* | AY879806 | AY879857 |  |
|  | *Centaurium bianoris* | AY879809 | AY879860 |  |
|  | *Centaurium cachanlahuen* | AY251695 | AY251725 |  |
|  | *Centaurium capense* |  | AY047874 |  |
|  | *Centaurium capitatum* | Z48114 | Z48141 |  |
|  | *Centaurium centaurioides* | AY879810 | AY879861 |  |
|  | *Centaurium chloodes* | AY879811 | AY879862 |  |
|  | *Centaurium erythraea* | AY879816 | AY879867 |  |
|  | *Centaurium favargeri* | AY251670 | AY251700 |  |
|  | *Centaurium gypsicola* | AY251671 | AY251701 |  |
|  | *Centaurium littorale* | AY879823 | AY879874 |  |
|  | *Centaurium mairei* | AY879825 | AY879876 |  |
|  | *Centaurium majus* | AY879827 | AY879878 |  |
|  | *Centaurium malzacianum* | AY879832 | AY879883 |  |
|  | *Centaurium maritimum* | AY879834 | AY879885 |  |
|  | *Centaurium pulchellum* | AY879836 | AY879887 |  |
|  | *Centaurium scilloides* | AY879840 | AY879891 |  |
|  | *Centaurium serpentinicola* | AY879841 | AY879892 |  |
|  | *Centaurium somedanum* | AY879842 | AY879893 |  |
|  | *Centaurium suffruticosum* | AY879845 | AY879896 |  |
|  | *Centaurium tenuiflorum* | AY879855 | AY879906 |  |
|  | *Centaurium turcicum* | AY879853 | AY879904 |  |
|  | *Centaurium uliginosum* | AY879854 | AY879905 |  |
|  | *Chironia baccifera* | AY251690 | AY251720 |  |
|  | *Chironia laxa* | AY251691 | AY251721 |  |
|  | *Chironia linoides* | AY251692 | AY251722 |  |
|  | *Cicendia filiformis* | AJ011463 | AJ011473 |  |
|  | *Cicendia quadrangularis* | AY251682 | AY251712 |  |
|  | *Eustoma exaltatum* | AY251698 | AY251728 |  |
|  | *Eustoma grandiflorum* | AY251696 | AY251726 |  |
|  | *Exaculum pusillum* | AY251681 | AY251711 |  |
|  | *Gyrandra brachycalyx* | AY047771 | AY047856 |  |
|  | *Gyrandra tenuifolia* |  | AY047857 |  |
|  | *Ixanthus viscosus* | AY251683 | AY251713 |  |
|  | *Orphium frutescens* | AY251693 | AY251723 |  |
|  | *Sabatia angularis* | AJ011467 | AJ011477 |  |
|  | *Sabatia campestris* | AY256382 | AY256387 |  |
|  | *Sabatia dodecandra* | AY256383 | AY256388 |  |
|  | *Sabatia stellaris* | AY256384 | AY256389 |  |
|  | *Schenkia australis* | AY251679 | AY251709 |  |
|  | *Schenkia clementii* | AY251680 | AY251710 |  |
|  | *Schenkia spicata* | AY047792 | AY047877 |  |
|  | *Zeltnera abramsii* | AY047712 | AY047797 |  |
|  | *Zeltnera arizonica* | AY047725 | AY047810 |  |
|  | *Zeltnera beyrichii* | AY047733 | AY047818 |  |
|  | *Zeltnera breviflora* | AY047736 | AY047821 |  |
|  | *Zeltnera calycosa* | AY047741 | AY047826 |  |
|  | *Zeltnera exaltata* | AY047723 | AY047808 |  |
|  | *Zeltnera glandulifera* | AY047731 | AY047816 |  |
|  | *Zeltnera madrensis* | AY047742 | AY047827 |  |
|  | *Zeltnera martinii* | AY047750 | AY047835 |  |
|  | *Zeltnera maryanna* | AY047730 | AY047815 |  |
|  | *Zeltnera muehlenbergii* | AY315668 | AY315673 |  |
|  | *Zeltnera multicaulis* | AY047728 | AY047813 |  |
|  | *Zeltnera namophila* | AY047711 | AY047796 |  |
|  | *Zeltnera nevadensis* | AY047719 | AY047804 |  |
|  | *Zeltnera nudicaulis* | AY047745 | AY047830 |  |
|  | *Zeltnera pusilla* | AY047756 | AY047841 |  |
|  | *Zeltnera quitensis* | AY047769 | AY047854 |  |
|  | *Zeltnera setacea* | AY047748 | AY047833 |  |
|  | *Zeltnera stricta* | AY047759 | AY047844 |  |
|  | *Zeltnera texensis* | AY047735 | AY047820 |  |
|  | *Zeltnera trichantha* | AY047710 | AY047795 |  |
|  | *Zeltnera venusta* | AY047715 | AY047800 |  |
|  | *Zeltnera wigginsii* | AY047754 | AY047839 |  |
|  | Outgroup: *Microrphium pubescens* |  |  | AJ489916 |
| *Ericameria* |  |  |  |  |
|  | *Ericameria albida* |  |  | AY170950 |
|  | *Ericameria arborescens* |  |  | AY170951 |
|  | *Ericameria bloomeri* |  |  | AY171006 |
|  | *Ericameria brachylepis* |  |  | AY171007 |
|  | *Ericameria cervina* |  |  | AY171009 |
|  | *Ericameria compacta* |  |  | AY171010 |
|  | *Ericameria cooperi* |  |  | AF046973 |
|  | *Ericameria crispa* |  |  | AY171011 |
|  | *Ericameria cuneata* |  |  | AF477641 |
|  | *Ericameria depressa* |  |  | AY170934 |
|  | *Ericameria discoidea* |  |  | AY171012 |
|  | *Ericameria eremobia* |  |  | AY170935 |
|  | *Ericameria ericoides* |  |  | AF477642 |
|  | *Ericameria fasciculata* |  |  | AY171014 |
|  | *Ericameria filifolia* |  |  | AY170938 |
|  | *Ericameria gilmanii* |  |  | AY171015 |
|  | *Ericameria graminea* |  |  | AY170936 |
|  | *Ericameria greenei* |  |  | AY171016 |
|  | *Ericameria humilis* |  |  | AY170939 |
|  | *Ericameria juarezensis* |  |  | AY171017 |
|  | *Ericameria laricifolia* |  |  | AY171018 |
|  | *Ericameria lignumviridis* |  |  | AY171019 |
|  | *Ericameria linearifolia* |  |  | AY171020 |
|  | *Ericameria linifolia* |  |  | AY170940 |
|  | *Ericameria martirensis* |  |  | AY171021 |
|  | *Ericameria molesta* |  |  | AY170941 |
|  | *Ericameria nana* |  |  | AY171022 |
|  | *Ericameria nauseosa* |  |  | AY171023 |
|  | *Ericameria obovata* |  |  | AY171024 |
|  | *Ericameria ophitidis* |  |  | AY171025 |
|  | *Ericameria palmeri* |  |  | AY171026 |
|  | *Ericameria paniculata* |  |  | AY170953 |
|  | *Ericameria parishii* |  |  | AY171028 |
|  | *Ericameria parryi* |  |  | AY171029 |
|  | *Ericameria pinifolia* |  |  | AY171030 |
|  | *Ericameria pulchella* |  |  | AY170942 |
|  | *Ericameria resinosa* |  |  | AY171031 |
|  | *Ericameria spathulata* |  |  | AY170943 |
|  | *Ericameria suffruticosa* |  |  | AY171032 |
|  | *Ericameria teretifolia* |  |  | AY170954 |
|  | *Ericameria vaseyi* |  |  | AY170944 |
|  | *Ericameria viscidiflora* |  |  | AY170947 |
|  | *Ericameria watsonii* |  |  | AY171034 |
|  | *Ericameria zionis* |  |  | AY171035 |
|  | Outgroup: *Chrysoma pauciflosculosa* |  |  | AY523843 |
| *Lepidium* |  |  |  |  |
|  | *Lepidium affghanum* |  |  | DQ780948 |
|  | *Lepidium africanum* | AJ582441 | AJ582498 |  |
|  | *Lepidium aletes* | FM178548 | FM178549 |  |
|  | *Lepidium alluaudii* | AJ582436 | AJ582493 |  |
|  | *Lepidium apetalum* |  |  | FJ980405 |
|  | *Lepidium arbuscula* | AJ582451 | AJ582517 |  |
|  | *Lepidium armoracia* | AJ582454 | AJ582502 |  |
|  | *Lepidium aschersonii* | AJ582426 | AJ582483 |  |
|  | *Lepidium aucheri* | AJ582443 | AJ582525 |  |
|  | *Lepidium austrinum* | AJ582467 | AJ582515 |  |
|  | *Lepidium banksii* | AJ582433 | AJ582490 |  |
|  | *Lepidium bidentatum* | AJ582468 | AJ582516 |  |
|  | *Lepidium bipinnatifidum* | AJ582446 | AJ582522 |  |
|  | *Lepidium biplicatum* | FM178550 | FM178551 |  |
|  | *Lepidium bonariense* | AJ582458 | AJ582506 |  |
|  | *Lepidium botschantsevianum* | AM991789 | AM991790 |  |
|  | *Lepidium brachyotum* | AM991791 | AM991792 |  |
|  | *Lepidium campestre* | AJ582412 | AJ582469 |  |
|  | *Lepidium capense* | AJ582452 | AJ582500 |  |
|  | *Lepidium capitatum* | FM178552 | FM178553 |  |
|  | *Lepidium cardamine* | FM178554 | FM178555 |  |
|  | *Lepidium cartilagineum* | AM991703 | AM991704 |  |
|  | *Lepidium chalepense* | AM991773 | AM991774 |  |
|  | *Lepidium chichicara* | AM991705 | AM991706 |  |
|  | *Lepidium cordatum* | AM991707 | AM991708 |  |
|  | *Lepidium coronopifolium* | AM991709 | AM991710 |  |
|  | *Lepidium costaricense* | AM991711 | AM991712 |  |
|  | *Lepidium danielsii* | AM991713 | AM991714 |  |
|  | *Lepidium davisii* |  |  | FJ541494 |
|  | *Lepidium depressum* | AM991717 | AM991718 |  |
|  | *Lepidium desertorum* | AJ582453 | AJ582501 |  |
|  | *Lepidium desvauxii* | AJ582429 | AJ582486 |  |
|  | *Lepidium dictyotum* | AJ582415 | AJ582472 |  |
|  | *Lepidium didymum* | AM991775 | AM991776 |  |
|  | *Lepidium divaricatum* | AJ582437 | AJ582494 |  |
|  | *Lepidium draba* | FM164554 | FM164555 |  |
|  | *Lepidium echinatum* | AM991719 | AM991720 |  |
|  | *Lepidium englerianum* | AM991777 | AM991778 |  |
|  | *Lepidium fasciculatum* | AJ582428 | AJ582485 |  |
|  | *Lepidium ferganense* | AJ582449 | AJ582519 |  |
|  | *Lepidium flavum* | AJ582444. | AJ582524 |  |
|  | *Lepidium flexicaule* | AJ582430 | AJ582487 |  |
|  | *Lepidium fremontii* | AJ582456 | AJ582504 |  |
|  | *Lepidium graminifolium* | AM991721 | AM991722 |  |
|  | *Lepidium heterophyllum* | AM991693 | AM991694 |  |
|  | *Lepidium hirtum* | AM991695 | AM991696 |  |
|  | *Lepidium hyssopifolium* | AJ582435 | AJ582492 |  |
|  | *Lepidium jaredii* | AM991733 | AM991734 |  |
|  | *Lepidium johnstonii* | AM991735 | AM991736 |  |
|  | *Lepidium kirkii* | EF109738 | EF109739 |  |
|  | *Lepidium lasiocarpum* | AJ582455 | AJ582503 |  |
|  | *Lepidium latifolium* | AJ582447 | AJ582521 |  |
|  | *Lepidium latipes* | AJ582416 | AJ582473 |  |
|  | *Lepidium leptopetalum* | AM991737 | AM991738 |  |
|  | *Lepidium linifolium* | AM991739 | AM991740 |  |
|  | *Lepidium litwinowii* | AM991795 | AM991796 |  |
|  | *Lepidium lyratum* | AJ582448 | AJ582520 |  |
|  | *Lepidium meyenii* | AJ582445 | AJ582523 |  |
|  | *Lepidium montanum* |  |  | EF367970 |
|  | *Lepidium morissonii* | AM991741 | AM991742 |  |
|  | *Lepidium muelleri-ferdinandi* | AJ582427 | AJ582484 |  |
|  | *Lepidium myriocarpum* | AJ582442 | AJ582499 |  |
|  | *Lepidium naufragorum* | AJ582422 | AJ582479 |  |
|  | *Lepidium navasii* | AM991779 | AM991780 |  |
|  | *Lepidium nitidum* | AJ582414 | AJ582471 |  |
|  | *Lepidium oblongum* | AJ582462 | AJ582510 |  |
|  | *Lepidium oleraceum* | AJ582434 | AJ582491 |  |
|  | *Lepidium orbiculare* | AM991743 | AM991744 |  |
|  | *Lepidium oxycarpum* | AJ582417 | AJ582474 |  |
|  | *Lepidium oxytrichum* | AJ582424 | AJ582481 |  |
|  | *Lepidium paniculatum* | FM164556 | FM164557 |  |
|  | *Lepidium papilliferum* |  |  | FJ541498 |
|  | *Lepidium papillosum* | AJ582425 | AJ582482 |  |
|  | *Lepidium pedicellosum* | AM991745 | AM991746 |  |
|  | *Lepidium perfoliatum* |  |  | DQ399120 |
|  | *Lepidium phlebopetalum* | FM178556 | FM178557 |  |
|  | *Lepidium pholidogynum* | AM991749 | AM991750 |  |
|  | *Lepidium pinnatifidum* | AJ582464 | AJ582512 |  |
|  | *Lepidium pinnatum* | AJ582439 | AJ582496 |  |
|  | *Lepidium platypetalum* |  |  | DQ780949 |
|  | *Lepidium pseudohyssopifolium* | AJ582431 | AJ582488 |  |
|  | *Lepidium pseudopapillosum* | AJ582423 | AJ582480 |  |
|  | *Lepidium pseudotasmanicum* | AJ582432 | AJ582489 |  |
|  | *Lepidium quitense* | AJ582463 | AJ582511 |  |
|  | *Lepidium rahmeri* | AM991751 | AM991752 |  |
|  | *Lepidium ramosissimum* | AM991753 | AM991754 |  |
|  | *Lepidium rhytidocarpum* | AM991783 | AM991784 |  |
|  | *Lepidium rigidum* | AM991755 | AM991756 |  |
|  | *Lepidium rotundum* |  |  | DQ780950 |
|  | *Lepidium ruderale* | AJ582465 | AJ582513 |  |
|  | *Lepidium sagittatum* | AM991799 | AM991800 |  |
|  | *Lepidium sativum* |  |  | AY662279 |
|  | *Lepidium schinzii* | AJ582440 | AJ582497 |  |
|  | *Lepidium serra* | AJ582450 | AJ582518 |  |
|  | *Lepidium serratum* | AM991785 | AM991786 |  |
|  | *Lepidium sisymbrioides* |  |  | DQ997570 |
|  | *Lepidium solandri* |  |  | DQ997571 |
|  | *Lepidium solomonii* | AM991757 | AM991758 |  |
|  | *Lepidium songaricum* | AM991759 | AM991760 |  |
|  | *Lepidium spinescens* | AJ582461 | AJ582509 |  |
|  | *Lepidium spinosum* | AJ582460 | AJ582508 |  |
|  | *Lepidium strictum* | AM991761 | AM991762 |  |
|  | *Lepidium subulatum* | AM991763 | AM991764 |  |
|  | *Lepidium tayloriae* | AM991765 | AM991766 |  |
|  | *Lepidium tenuicaule* | AJ582421 | AJ582478 |  |
|  | *Lepidium thrurberi* | AM991767 | AM991768 |  |
|  | *Lepidium tiehmii* | FM164558 | FM164559 |  |
|  | *Lepidium trifurcum* | AJ582438 | AJ582495 |  |
|  | *Lepidium vesicarium* | AM991769 | AM991770 |  |
|  | *Lepidium villarsii* | AM991771 | AM991772 |  |
|  | *Lepidium virginicum* |  |  | AY662280 |
|  | Outgroup: *Hornungia petraea* | FM164548 | FM164549 |  |
| *Lotus* |  |  |  |  |
|  | *Lotus aboriginus* |  |  | AF218522 |
|  | *Lotus aegaeus* |  |  | DQ160276 |
|  | *Lotus alpinus* |  |  | DQ160274 |
|  | *Lotus angustissimus* |  |  | DQ166243 |
|  | *Lotus arabicus* |  |  | AF450176 |
|  | *Lotus arenarius* |  |  | AF450193 |
|  | *Lotus argophyllus* |  |  | AF218515 |
|  | *Lotus assakensis* |  |  | DQ160277 |
|  | *Lotus australis* |  |  | AF450187 |
|  | *Lotus azoricus* |  |  | AY294293 |
|  | *Lotus benoistii* | DQ372916 |  |  |
|  | *Lotus benthamii* |  |  | AF450203 |
|  | *Lotus berthelotii* |  |  | AY294306 |
|  | *Lotus borbasii* |  |  | DQ166226 |
|  | *Lotus broussonetii* |  |  | DQ160278 |
|  | *Lotus campylocladus* |  |  | AF450196 |
|  | *Lotus castellanus* |  |  | DQ166238 |
|  | *Lotus cedrosensis* |  |  | AF450173 |
|  | *Lotus chihuahuanus* |  |  | AF450159 |
|  | *Lotus collinus* |  |  | AF450198 |
|  | *Lotus conimbricensis* |  |  | AF450186 |
|  | *Lotus corniculatus* |  |  | DQ312207 |
|  | *Lotus crassifolius* |  |  | AF450171 |
|  | *Lotus creticus* |  |  | DQ160279 |
|  | *Lotus cruentus* |  |  | AF450182 |
|  | *Lotus cytisoides* |  |  | DQ166241 |
|  | *Lotus delortii* |  |  | DQ166228 |
|  | *Lotus dendroideus* |  |  | AF450164 |
|  | *Lotus discolor* |  |  | DQ160288 |
|  | *Lotus distichus* |  |  | AF450199 |
|  | *Lotus dumetorum* |  |  | AY294294 |
|  | *Lotus edulis* |  |  | DQ311972 |
|  | *Lotus emeroides* |  |  | AY294295 |
|  | *Lotus eremiticus* |  |  | AY294307 |
|  | *Lotus eriosolen* |  |  | DQ160281 |
|  | *Lotus erythrorhizus* |  |  | AY294296 |
|  | *Lotus formosissimus* |  |  | AF450166 |
|  | *Lotus garcinii* |  |  | DQ166234 |
|  | *Lotus gebelia* |  |  | AF450188 |
|  | *Lotus glaber* |  |  | DQ166225 |
|  | *Lotus glaucus* |  |  | DQ311973 |
|  | *Lotus glinoides* |  |  | DQ166220 |
|  | *Lotus goetzei* |  |  | DQ166235 |
|  | *Lotus grandiflorus* |  |  | AF450163 |
|  | *Lotus halophilus* |  |  | DQ160283 |
|  | *Lotus hamatus* |  |  | AF450161 |
|  | *Lotus heermannii* |  |  | AF450165 |
|  | *Lotus hillebrandii* |  |  | AY294298 |
|  | *Lotus jacobaeus* |  |  | AY294299 |
|  | *Lotus japonicus* |  |  | DQ311975 |
|  | *Lotus jolyi* |  |  | DQ166240 |
|  | *Lotus krylovii* |  |  | AF450209 |
|  | *Lotus lalambensis* |  |  | DQ166216 |
|  | *Lotus lancerottensis* |  |  | AY294300 |
|  | *Lotus lanuginosus* |  |  | DQ166221 |
|  | *Lotus laricus* |  |  | DQ166233 |
|  | *Lotus leptophyllus* |  |  | AY294301 |
|  | *Lotus maculatus* |  |  | AY294308 |
|  | *Lotus maroccanus* |  |  | AF450181 |
|  | *Lotus mascaensis* |  |  | AY294302 |
|  | *Lotus michauxianus* |  |  | AF450206 |
|  | *Lotus mlanjeanus* |  |  | DQ166232 |
|  | *Lotus nevadensis* |  |  | AF450169 |
|  | *Lotus nuttallianus* |  |  | AF450179 |
|  | *Lotus oblongifolius* |  |  | AF450170 |
|  | *Lotus ononopsis* |  |  | DQ166219 |
|  | *Lotus ornithopodioides* |  |  | AF450205 |
|  | *Lotus oroboides* |  |  | AF450172 |
|  | *Lotus palustris* |  |  | AF450195 |
|  | *Lotus parviflorus* |  |  | DQ166230 |
|  | *Lotus peczoricus* |  |  | AF450191 |
|  | *Lotus pedunculatus* |  |  | DQ166222 |
|  | *Lotus peregrinus* |  |  | AF450177 |
|  | *Lotus polyphyllus* |  |  | DQ160289 |
|  | *Lotus praetermissus* |  |  | DQ168370 |
|  | *Lotus preslii* |  |  | DQ166236 |
|  | *Lotus procumbens* |  |  | AF450168 |
|  | *Lotus pseudocreticus* |  |  | DQ160284 |
|  | *Lotus purpureus* |  |  | AY294303 |
|  | *Lotus purshianus* |  |  | AF467067 |
|  | *Lotus pyranthus* |  |  | AY294309 |
|  | *Lotus quinatus* |  |  | DQ166217 |
|  | *Lotus rigidus* |  |  | AF450178 |
|  | *Lotus roudairei* |  |  | AF450200 |
|  | *Lotus salsuginosus* |  |  | AF450167 |
|  | *Lotus schimperi* |  |  | DQ166218 |
|  | *Lotus schoelleri* |  |  | DQ166224 |
|  | *Lotus scoparius* |  |  | AF450175 |
|  | *Lotus simonae* |  |  | DQ160285 |
|  | *Lotus spartioides* |  |  | AY294304 |
|  | *Lotus stepposus* |  |  | DQ166242 |
|  | *Lotus strictus* |  |  | DQ160286 |
|  | *Lotus strigosus* |  |  | AF450201 |
|  | *Lotus suaveolens* |  |  | DQ311978 |
|  | *Lotus subbiflorus* |  |  | DQ168369 |
|  | *Lotus subpinnatus* |  |  | AF450207 |
|  | *Lotus tenella* |  |  | AY294305 |
|  | *Lotus tetragonolobus* |  |  | AF450225 |
|  | *Lotus trigonelloides* |  |  | AF450202 |
|  | *Lotus uliginosus* |  |  | DQ160273 |
|  | *Lotus unifoliolatus* |  |  | DQ311969 |
|  | *Lotus weilleri* |  |  | AF450180 |
|  | *Lotus wildii* |  |  | DQ160287 |
|  | *Lotus wrangelianus* |  |  | AF450174 |
|  | Outgroup: *Vicia oroboides* |  |  | AM950283 |
| *Lupinus* |  |  |  |  |
|  | *Lupinus affinis* |  |  | AF007487 |
|  | *Lupinus albescens* |  |  | DQ524190 |
|  | *Lupinus albifrons* | Z72164 | Z72165 |  |
|  | *Lupinus albus* |  |  | DQ524191 |
|  | *Lupinus anatolicus* |  |  | AF108085 |
|  | *Lupinus andersonii* |  |  | AY338934 |
|  | *Lupinus angustifolius* |  |  | DQ524193 |
|  | *Lupinus arboreus* |  |  | DQ524196 |
|  | *Lupinus arcticus* | Z72156 | Z72157 |  |
|  | *Lupinus argenteus* |  |  | DQ524197 |
|  | *Lupinus aridus* | AF007446 | AF007447 |  |
|  | *Lupinus arizonicus* |  |  | DQ524198 |
|  | *Lupinus arvensis* |  |  | DQ524199 |
|  | *Lupinus aschenbornii* | Z72190 | Z72191 |  |
|  | *Lupinus atlanticus* | AF007432 | AF007433 |  |
|  | *Lupinus aureonitens* | Z72210 | Z72211 |  |
|  | *Lupinus ballianus* |  |  | DQ524201 |
|  | *Lupinus bandelierae* |  |  | DQ524206 |
|  | *Lupinus bangii* |  |  | DQ524300 |
|  | *Lupinus benthamii* | Z72168 | Z72169 |  |
|  | *Lupinus bicolor* |  |  | DQ524209 |
|  | *Lupinus bogotensis* | Z72192 | Z72193 |  |
|  | *Lupinus bracteolaris* |  |  | DQ524211 |
|  | *Lupinus brevicaulis* |  |  | DQ524213 |
|  | *Lupinus breweri* |  |  | AY338938 |
|  | *Lupinus chachas* |  |  | DQ524216 |
|  | *Lupinus chamissonis* |  |  | DQ524219 |
|  | *Lupinus chrysanthus* |  |  | DQ524221 |
|  | *Lupinus concinnus* |  |  | DQ524214 |
|  | *Lupinus cosentinii* |  |  | DQ524222 |
|  | *Lupinus crotalarioides* |  |  | DQ524225 |
|  | *Lupinus cruckshanskii* | Z72194 | Z72195 |  |
|  | *Lupinus cumulicola* |  |  | DQ524227 |
|  | *Lupinus diffusus* | AY609190 | AY609191 |  |
|  | *Lupinus digitatus* | AF007430 | AF007431 |  |
|  | *Lupinus duranii* |  |  | AF007493 |
|  | *Lupinus elegans* | AF007462 | AF007463 |  |
|  | *Lupinus ellsworthianus* |  |  | DQ524215 |
|  | *Lupinus excubitus* |  |  | AF007492 |
|  | *Lupinus formosus* | Z72172 | Z72173 |  |
|  | *Lupinus gibertianus* |  |  | DQ524229 |
|  | *Lupinus grayi* |  |  | AY338939 |
|  | *Lupinus guaraniticus* |  |  | DQ524231 |
|  | *Lupinus havardii* |  |  | DQ524233 |
|  | *Lupinus hirsutissimus* |  |  | DQ524235 |
|  | *Lupinus hispanicus* |  |  | DQ524236 |
|  | *Lupinus huaronensis* |  |  | DQ524237 |
|  | *Lupinus huigrensis* |  |  | DQ524304 |
|  | *Lupinus jaime-hintonianus* | AY609184 | AY609197 |  |
|  | *Lupinus lanatus* |  |  | DQ524242 |
|  | *Lupinus latifolius* |  |  | DQ524243 |
|  | *Lupinus lepidus* |  |  | DQ524246 |
|  | *Lupinus leucophyllus* | AF007442 | AF007443 |  |
|  | *Lupinus lindleyanus* |  |  | DQ524247 |
|  | *Lupinus linearis* |  |  | DQ524248 |
|  | *Lupinus littoralis* | AF007452 | AF007453 |  |
|  | *Lupinus luteolus* |  |  | AF007490 |
|  | *Lupinus luteus* |  |  | DQ524249 |
|  | *Lupinus magnistipulatus* |  |  | DQ524251 |
|  | *Lupinus mantaroensis* |  |  | DQ524200 |
|  | *Lupinus mexicanus* | AF007444 | AF007445 |  |
|  | *Lupinus micranthus* |  |  | DQ524252 |
|  | *Lupinus microcarpus* |  |  | DQ524253 |
|  | *Lupinus microphyllus* |  |  | DQ524255 |
|  | *Lupinus minimus* |  |  | AF007497 |
|  | *Lupinus misticola* |  |  | DQ524257 |
|  | *Lupinus mollendoensis* |  |  | DQ524258 |
|  | *Lupinus montanus* |  |  | DQ524259 |
|  | *Lupinus multiflorus* |  |  | DQ524260 |
|  | *Lupinus mutabilis* |  |  | DQ524273 |
|  | *Lupinus nanus* | AF007440 | AF007441 |  |
|  | *Lupinus neomexicanus* |  |  | DQ524274 |
|  | *Lupinus nootkatensis* | Z72160 | Z72161 |  |
|  | *Lupinus nubigenus* |  |  | DQ524275 |
|  | *Lupinus odoratus* |  |  | DQ524276 |
|  | *Lupinus palaestinus* |  |  | AF007479 |
|  | *Lupinus paraguayensis* | Z72214 | Z72215 |  |
|  | *Lupinus paranensis* |  |  | DQ524279 |
|  | *Lupinus parvifolius* |  |  | DQ524280 |
|  | *Lupinus perennis* | Z72162 | Z72163 |  |
|  | *Lupinus pilosus* | AF007434 | AF007435 |  |
|  | *Lupinus piurensis* |  |  | DQ524284 |
|  | *Lupinus polycarpus* | Z72180 | Z72181 |  |
|  | *Lupinus polyphyllus* | Z72154 | Z72155 |  |
|  | *Lupinus praestabilis* |  |  | DQ524285 |
|  | *Lupinus princei* | Z72224 | Z72225 |  |
|  | *Lupinus prostratus* |  |  | DQ524286 |
|  | *Lupinus pubescens* | Z72178 | Z72179 |  |
|  | *Lupinus pulvinaris* |  |  | DQ524288 |
|  | *Lupinus purosericeus* |  |  | DQ524238 |
|  | *Lupinus pusillus* |  |  | AF007491 |
|  | *Lupinus ramosissimus* |  |  | DQ524289 |
|  | *Lupinus reitzii* |  |  | DQ524290 |
|  | *Lupinus rivularis* | Z72182 | Z72183 |  |
|  | *Lupinus rubiflorus* |  |  | DQ524292 |
|  | *Lupinus sarmentosus* |  |  | DQ524291 |
|  | *Lupinus semperflorens* |  |  | DQ524297 |
|  | *Lupinus sericeus* | AF007436 | AF007437 |  |
|  | *Lupinus sierrae-blancae* |  |  | DQ524298 |
|  | *Lupinus solanagrorum* |  |  | DQ524294 |
|  | *Lupinus sparsiflorus* |  |  | AF007482 |
|  | *Lupinus subacaulis* |  |  | DQ524312 |
|  | *Lupinus subsessilis* |  |  | DQ524313 |
|  | *Lupinus succulentus* | Z72184 | Z72185 |  |
|  | *Lupinus sulphureus* | AF007460 | AF007461 |  |
|  | *Lupinus tatapacensis* |  |  | DQ524314 |
|  | *Lupinus texensis* |  |  | DQ524316 |
|  | *Lupinus tomentosus* |  |  | DQ524317 |
|  | *Lupinus tominensis* |  |  | DQ524207 |
|  | *Lupinus truncatus* |  |  | DQ524318 |
|  | *Lupinus uleanus* |  |  | DQ524319 |
|  | *Lupinus velutinus* |  |  | DQ524321 |
|  | *Lupinus villosus* | AY609189 | AY609192 |  |
|  | *Lupinus weberbaueri* |  |  | DQ524324 |
|  | *Lupinus wyethii* |  |  | AY949016 |
|  | Outgroup: *Genista umbellata* |  |  | DQ524184 |
| Lycieae |  |  |  |  |
|  | *Grabowskia boerhaviifolia* |  |  | FJ439754 |
|  | *Grabowskia duplicata* |  |  | AF238982 |
|  | *Grabowskia glauca* | AB019289 | AB019949 |  |
|  | *Grabowskia obtusa* |  |  | FJ439755 |
|  | *Lycium ameghinoi* |  |  | DQ124618 |
|  | *Lycium americanum* |  |  | DQ124619 |
|  | *Lycium andersonii* |  |  | DQ124620 |
|  | *Lycium arenicola* |  |  | FJ439756 |
|  | *Lycium athium* |  |  | FJ439757 |
|  | *Lycium australe* | AY028131 | AY028149 |  |
|  | *Lycium barbarum* | AY028132 | AY028150 |  |
|  | *Lycium berlandieri* |  |  | AF238989 |
|  | *Lycium brevipes* |  |  | DQ124621 |
|  | *Lycium californicum* |  |  | DQ124653 |
|  | *Lycium carolinianum* | AY028133 | AY028151 |  |
|  | *Lycium cestroides* |  |  | DQ124623 |
|  | *Lycium chilense* | AY028137 | AY028155 |  |
|  | *Lycium ciliatum* | AY028136 | AY028154 |  |
|  | *Lycium cooperi* |  |  | AF238984 |
|  | *Lycium cuneatum* |  |  | FJ439758 |
|  | *Lycium elongatum* |  |  | DQ124624 |
|  | *Lycium exsertum* |  |  | DQ124625 |
|  | *Lycium ferocissimum* | AY028139 | AY028157 |  |
|  | *Lycium fremontii* |  |  | DQ124626 |
|  | *Lycium infaustum* |  |  | DQ124627 |
|  | *Lycium leiospermum* |  |  | FJ439759 |
|  | *Lycium macrodon* |  |  | DQ124628 |
|  | *Lycium morongii* |  |  | DQ124629 |
|  | *Lycium nodosum* |  |  | DQ124630 |
|  | *Lycium pallidum* |  |  | DQ124631 |
|  | *Lycium parishii* |  |  | DQ124632 |
|  | *Lycium puberulum* |  |  | AF238985 |
|  | *Lycium rachidocladum* |  |  | FJ439760 |
|  | *Lycium sandwicense* | AY028142 | AY028160 |  |
|  | *Lycium shawii* | AY028143 | AY028161 |  |
|  | *Lycium shockleyi* |  |  | AF238987 |
|  | *Lycium tenue* | AY028144 | AY028162 |  |
|  | *Lycium tenuispinosum* |  |  | DQ124633 |
|  | *Lycium tetrandrum* | AY028145 | AY028163 |  |
|  | *Lycium texanum* |  |  | FJ439761 |
|  | *Lycium torreyi* |  |  | DQ124634 |
|  | *Lycium villosum* |  |  | FJ439762 |
|  | *Lycium vimineum* |  |  | DQ124635 |
|  | *Phrodus macrophyllus* |  |  | FJ439765 |
|  | Outgroup: *Nolana werdermannii* |  |  | FJ439764 |
| Phrymoideae |  |  |  |  |
|  | *Mimulus alsinoides* |  |  | AY575450 |
|  | *Mimulus ampliatus* |  |  | AY575421 |
|  | *Mimulus androsaceus* |  |  | AY575400 |
|  | *Mimulus angustatus* |  | AY575378 |  |
|  | *Mimulus auranticus* |  |  | AF478917 |
|  | *Mimulus bicolor* |  |  | AY575409 |
|  | *Mimulus bifidus* |  |  | AY575392 |
|  | *Mimulus bigelovii* |  |  | AY575353 |
|  | *Mimulus bodinieri* |  |  | AY575434 |
|  | *Mimulus bolanderi* |  |  | AY575352 |
|  | *Mimulus breviflorus* |  |  | AY575425 |
|  | *Mimulus brevipes* |  |  | AY575358 |
|  | *Mimulus cardinalis* |  |  | AY575414 |
|  | *Mimulus clevelandii* |  |  | AY575386 |
|  | *Mimulus clivicola* |  |  | AY575369 |
|  | *Mimulus congdonii* |  | AY575383 |  |
|  | *Mimulus constrictus* |  |  | AY575349 |
|  | *Mimulus cupreus* |  |  | AY575447 |
|  | *Mimulus cusickii* |  |  | AY575368 |
|  | *Mimulus dentatus* |  |  | AY575449 |
|  | *Mimulus dentilobus* |  |  | AY575444 |
|  | *Mimulus depressus* |  |  | AY575446 |
|  | *Mimulus douglasii* |  |  | AY575381 |
|  | *Mimulus dudleyi* |  |  | AY575432 |
|  | *Mimulus eastwoodiae* |  |  | AY575417 |
|  | *Mimulus evanescens* |  |  | AY575455 |
|  | *Mimulus exiguus* |  |  | AY575420 |
|  | *Mimulus filicaulis* |  |  | AY575410 |
|  | *Mimulus flemingii* |  |  | AY575395 |
|  | *Mimulus floribundus* |  |  | AY575431 |
|  | *Mimulus fremontii* |  |  | AY575357 |
|  | *Mimulus gemmiparus* |  |  | AY575451 |
|  | *Mimulus glabratus* |  |  | AY575445 |
|  | *Mimulus glaucescens* |  |  | AY575443 |
|  | *Mimulus gracilipes* |  |  | AY575407 |
|  | *Mimulus gracilis* |  |  | AF478934 |
|  | *Mimulus guttatus* |  |  | AY575439 |
|  | *Mimulus hymenophyllus* |  |  | AY575422 |
|  | *Mimulus inconspicuus* |  |  | AY575452 |
|  | *Mimulus jepsonii* |  |  | AY575370 |
|  | *Mimulus johnstonii* |  |  | AY575359 |
|  | *Mimulus jungermannioides* |  |  | AY575426 |
|  | *Mimulus kelloggii* |  |  | AY575382 |
|  | *Mimulus latidens* |  |  | AY575429 |
|  | *Mimulus layneae* |  |  | AY575372 |
|  | *Mimulus leptaleus* |  |  | AY575351 |
|  | *Mimulus lewisii* |  |  | AY575416 |
|  | *Mimulus longiflorus* |  |  | AY575394 |
|  | *Mimulus luteus* |  |  | AY575448 |
|  | *Mimulus mephiticus* |  |  | AY575355 |
|  | *Mimulus micranthus* |  |  | AY575440 |
|  | *Mimulus mohavensis* |  |  | AY575375 |
|  | *Mimulus montioides* |  |  | AY575405 |
|  | *Mimulus moschatus* |  |  | AY575428 |
|  | *Mimulus nanus* |  |  | AY575366 |
|  | *Mimulus nelsonii* |  |  | AY575413 |
|  | *Mimulus nepalensis* |  |  | AY575435 |
|  | *Mimulus norrisii* |  |  | AY575433 |
|  | *Mimulus nudatus* |  |  | AY575441 |
|  | *Mimulus palmeri* |  |  | AY575406 |
|  | *Mimulus parishii* |  |  | AY575411 |
|  | *Mimulus parryi* |  |  | AY575385 |
|  | *Mimulus patulus* |  |  | AY575423 |
|  | *Mimulus pictus* |  |  | AY575376 |
|  | *Mimulus primuloides* |  |  | AY575419 |
|  | *Mimulus prostratus* |  |  | AY943125 |
|  | *Mimulus pulchellus* |  |  | AY575380 |
|  | *Mimulus pulsiferae* |  |  | AY575424 |
|  | *Mimulus puniceus* |  |  | AY575388 |
|  | *Mimulus purpureus* |  |  | AY575402 |
|  | *Mimulus rattanii* |  |  | AY575356 |
|  | *Mimulus repens* |  |  | AY943117 |
|  | *Mimulus ringens* |  |  | AY575454 |
|  | *Mimulus rubellus* |  |  | AY575408 |
|  | *Mimulus rupestris* |  |  | AY575412 |
|  | *Mimulus rupicola* |  | AY575384 |  |
|  | *Mimulus shevockii* |  |  | AY575403 |
|  | *Mimulus suksdorfii* |  |  | AY575401 |
|  | *Mimulus szechuanensis* |  |  | FJ172743 |
|  | *Mimulus tenellus* |  |  | AY575436 |
|  | *Mimulus tilingii* |  |  | AY575442 |
|  | *Mimulus torreyi* |  |  | AY575374 |
|  | *Mimulus tricolor* |  | AY575377 |  |
|  | *Mimulus uvedaliae* |  |  | AF478936 |
|  | *Mimulus verbenaceus* |  |  | AY575418 |
|  | *Mimulus viscidus* |  | AY575354 |  |
|  | *Mimulus washingtonensis* |  |  | AY575427 |
|  | *Mimulus whitneyi* |  |  | AY575348 |
|  | *Mimulus wiensii* |  |  | AY575437 |
|  | *Mimulus yeocorensis* |  |  | AY575438 |
|  | *Berendtia rugosa* |  |  | AY575398 |
|  | *Elacholoma* sp. |  |  | AY943118 |
|  | *Glossostigma cleistanthum* |  |  | DQ371428 |
|  | *Glossostigma diandrum* |  |  | AY943121 |
|  | *Glossostigma drummondi* |  |  | AF478937 |
|  | *Glossostigma elatinoides* |  |  | AY943119 |
|  | *Hemichaena fruticosa* |  |  | AY575399 |
|  | *Leucocarpus perfoliatus* |  |  | AY575453 |
|  | *Peplidium aithocheilum* |  |  | AF478938 |
|  | *Peplidium foecundum* |  |  | AY943132 |
|  | *Peplidium maritimum* |  |  | AY943139 |
|  | *Peplidium meulleri* |  |  | AY943134 |
|  | *Phryma leptostachya* |  |  | AF478924 |
|  | Immediate outgroup: *Mazus spicatus* | |  | FJ172740 |
|  | Calibrated outgroup: *Verbina urticifolia* | |  | DQ006043 |
| Polemoniaceae |  |  |  |  |
|  | *Acanthogilia gloriosa* |  |  | EU339722 |
|  | *Aliciella caespitosa* |  |  | EF199698 |
|  | *Aliciella hutchinsifolia* |  |  | AF208193 |
|  | *Aliciella latifolia* |  |  | EU339745 |
|  | *Aliciella leptomeria* |  |  | AF208195 |
|  | *Aliciella mcvickerae* |  |  | EU339743 |
|  | *Aliciella subnuda* |  |  | EU628284 |
|  | *Aliciella tenuis* |  |  | EU628285 |
|  | *Aliciella triodon* |  |  | EU339744 |
|  | *Allophyllum divaricatum* |  |  | EU339730 |
|  | *Allophyllum gilioides* |  |  | AF202949 |
|  | *Allophyllum glutinosum* |  |  | EU339728 |
|  | *Allophyllum integrifolium* |  |  | EU339729 |
|  | *Bonplandia geminiflora* |  |  | EU339723 |
|  | *Bryantiella glutinosa* |  |  | EU339754 |
|  | *Bryantiella palmeri* |  |  | EU339755 |
|  | *Cantua buxifolia* |  |  | EU339724 |
|  | *Cantua flexuosa* |  |  | EU628248 |
|  | *Cantua quercifolia* |  |  | EU339725 |
|  | *Cantua volcanica* |  |  | EU339726 |
|  | *Cobaea aequatoriensis* |  |  | AH005699 |
|  | *Cobaea aschersoniana* |  |  | AH005694 |
|  | *Cobaea biaurita* |  |  | AH005685 |
|  | *Cobaea campanulata* |  |  | AH005700 |
|  | *Cobaea flava* |  |  | AH005701 |
|  | *Cobaea gracilis* |  |  | AH005696 |
|  | *Cobaea lutea* |  |  | AH005706 |
|  | *Cobaea minor* |  |  | AH005679 |
|  | *Cobaea pachysepala* |  |  | AH005691 |
|  | *Cobaea penduliflora* |  |  | EU628250 |
|  | *Cobaea pringlei* |  |  | AH005681 |
|  | *Cobaea rotundiflora* |  |  | AH005707 |
|  | *Cobaea scandens* |  |  | EU339727 |
|  | *Cobaea skutchii* |  |  | AH005693 |
|  | *Cobaea stipularis* |  |  | AH005683 |
|  | *Cobaea trianae* |  |  | AH005689 |
|  | *Collomia cavanillesii* |  |  | DQ196896 |
|  | *Collomia debilis* |  |  | EU628290 |
|  | *Collomia diversifolia* |  |  | AY997921 |
|  | *Collomia grandiflora* |  |  | DQ196907 |
|  | *Collomia heterophylla* |  |  | EU339732 |
|  | *Collomia linearis* |  |  | EU339733 |
|  | *Collomia macrocalyx* |  |  | DQ196901 |
|  | *Collomia mazama* |  |  | EU628289 |
|  | *Collomia rawsoniana* |  |  | EU339734 |
|  | *Collomia renacta* |  |  | DQ196900 |
|  | *Collomia tenella* |  |  | DQ196905 |
|  | *Collomia tinctoria* |  |  | DQ196890 |
|  | *Collomia tracyi* |  |  | AY997923 |
|  | *Collomia wilkenii* |  |  | DQ196913 |
|  | *Dayia grantii* |  |  | EU339751 |
|  | *Dayia scabra* |  |  | EU339750 |
|  | *Eriastrum densifolium* |  |  | EU628267 |
|  | *Eriastrum signatum* |  |  | EU628268 |
|  | *Eriastrum wilcoxii* |  |  | EU339765 |
|  | *Gilia achilleifolia* |  |  | AF006097 |
|  | *Gilia angelensis* |  |  | AF208202 |
|  | *Gilia brecciarum* |  |  | AF208203 |
|  | *Gilia campanulata* |  |  | AF208205 |
|  | *Gilia cana* |  |  | AF208204 |
|  | *Gilia cana triceps* |  |  | EU339742 |
|  | *Gilia capitata* |  |  | EU339740 |
|  | *Gilia clivorum* |  |  | AF202935 |
|  | *Gilia clokeyi* |  |  | AF202952 |
|  | *Gilia crassifolia* |  |  | AF202951 |
|  | *Gilia diegensis* |  |  | EF199706 |
|  | *Gilia laciniata* |  |  | EU339741 |
|  | *Gilia leptantha* |  |  | EF199705 |
|  | *Gilia millefoliata* |  |  | AF202946 |
|  | *Gilia multicaulis* |  |  | AF006103 |
|  | *Gilia nevinii* |  |  | AF202947 |
|  | *Gilia polyantha* |  |  | EU339797 |
|  | *Gilia scopulorum* |  |  | AF208209 |
|  | *Gilia sinuata* |  |  | EF199707 |
|  | *Gilia stellata* |  |  | AF208212 |
|  | *Gilia tricolor* |  |  | EF199704 |
|  | *Gilia valdiviensis* |  |  | AF202948 |
|  | *Gilia yorkii* |  |  | EF199703 |
|  | *Giliastrum foetidum* |  |  | EU339748 |
|  | *Giliastrum ludens* |  |  | EU339746 |
|  | *Giliastrum purpusii* |  |  | EU628282 |
|  | *Giliastrum rigidulum* |  |  | EU339747 |
|  | *Gymnosteris nudicaulis* |  |  | EU628258 |
|  | *Gymnosteris parvula* |  |  | EU339816 |
|  | *Ipomopsis aggregata* |  |  | EU628261 |
|  | *Ipomopsis arizonica* |  |  | EU339807 |
|  | *Ipomopsis congesta* |  |  | EU628262 |
|  | *Ipomopsis effusa* |  |  | EU339769 |
|  | *Ipomopsis gossypifera* |  |  | EU339773 |
|  | *Ipomopsis gunnisonii* |  |  | EU339776 |
|  | *Ipomopsis guttata* |  |  | EU339770 |
|  | *Ipomopsis havardii* |  |  | EU339753 |
|  | *Ipomopsis laxiflora* |  |  | EU339796 |
|  | *Ipomopsis longiflora* |  |  | EU339795 |
|  | *Ipomopsis macombii* |  |  | EU339799 |
|  | *Ipomopsis macrosiphon* |  |  | EU339805 |
|  | *Ipomopsis monticola* |  |  | EU339792 |
|  | *Ipomopsis multiflora* |  |  | EU339798 |
|  | *Ipomopsis pinnata* |  |  | EU339790 |
|  | *Ipomopsis polyantha* |  |  | EU339800 |
|  | *Ipomopsis polycladon* |  |  | EU339772 |
|  | *Ipomopsis pringlei* |  |  | EU339791 |
|  | *Ipomopsis pumila* |  |  | EU339775 |
|  | *Ipomopsis roseata* |  |  | EU339784 |
|  | *Ipomopsis rubra* |  |  | EU339801 |
|  | *Ipomopsis sancti-spiritus* |  |  | EU339804 |
|  | *Ipomopsis sonorae* |  |  | EU339752 |
|  | *Ipomopsis spicata* |  |  | EU339786 |
|  | *Ipomopsis tenuifolia* |  |  | EU339771 |
|  | *Ipomopsis tenuituba* |  |  | EU339806 |
|  | *Ipomopsis thurberi* |  |  | EU339789 |
|  | *Ipomopsis tridactyla* |  |  | EU339788 |
|  | *Ipomopsis wendtii* |  |  | EU339802 |
|  | *Ipomopsis wrightii* |  |  | EU339774 |
|  | *Langloisia setosissima* |  |  | EU628279 |
|  | *Lathrocasis tenerrima* |  |  | EU339739 |
|  | *Leptodactylon caespitosum* | AF119443 | AF119469 |  |
|  | *Leptodactylon jaegeri* |  |  | AF067542 |
|  | *Leptodactylon pungens* |  |  | AF067543 |
|  | *Leptodactylon watsonii* |  |  | AF067544 |
|  | *Leptosiphon acicularis* | AF119424 | AF119450 |  |
|  | *Leptosiphon ambiguus* | AF119442 | AF119468 |  |
|  | *Leptosiphon androsaceus* |  |  | AF264727 |
|  | *Leptosiphon aureus* | AF119434 | AF119460 |  |
|  | *Leptosiphon bicolor* |  |  | AF264725 |
|  | *Leptosiphon bolanderi* | AF119440 | AF119466 |  |
|  | *Leptosiphon breviculus* |  |  | AF067545 |
|  | *Leptosiphon ciliatus* |  |  | EU339821 |
|  | *Leptosiphon filipes* | AF119437 | AF119463 |  |
|  | *Leptosiphon floribundus* |  |  | AF167258 |
|  | *Leptosiphon grandiflorus* |  |  | AF027693 |
|  | *Leptosiphon harknessii* | AF119433 | AF119459 |  |
|  | *Leptosiphon jepsonii* |  |  | AF264726 |
|  | *Leptosiphon lemmonii* |  |  | AF027694 |
|  | *Leptosiphon liniflorus* |  |  | AF027695 |
|  | *Leptosiphon montanus* | AF119423 | AF119449 |  |
|  | *Leptosiphon nudatus* |  |  | AF027692 |
|  | *Leptosiphon nuttallii* |  |  | EU339822 |
|  | *Leptosiphon pachyphyllus* |  |  | AF067550 |
|  | *Leptosiphon parviflorus* |  |  | AF264728 |
|  | *Leptosiphon pygmeaus* | AF119438 | AF119464 |  |
|  | *Leptosiphon rattanii* | AF119441 | AF119467 |  |
|  | *Leptosiphon septentrionalis* | AF119432 | AF119458 |  |
|  | *Linanthus arenicola* | AF068832 | AF068833 |  |
|  | *Linanthus bellus* |  |  | AF027689 |
|  | *Linanthus bigelovii* |  |  | AF027690 |
|  | *Linanthus concinnus* |  |  | AF027702 |
|  | *Linanthus demissus* |  |  | EU339819 |
|  | *Linanthus dianthiflorus* |  |  | AF027697 |
|  | *Linanthus dichotomus* |  |  | EU339820 |
|  | *Linanthus filiformis* |  |  | EU339815 |
|  | *Linanthus inyoensis* |  |  | EU628260 |
|  | *Linanthus jaegeri* |  |  | EU339817 |
|  | *Linanthus jamauensis* | AF119436 | AF119462 |  |
|  | *Linanthus jonesii* | AF119430 | AF119456 |  |
|  | *Linanthus killipii* |  |  | AF027698 |
|  | *Linanthus laxus* | AF119427 | AF119453 |  |
|  | *Linanthus maculatus* |  |  | AY997926 |
|  | *Linanthus melingii* | AF119428 | AF119454 |  |
|  | *Linanthus minimus* |  |  | AF264729 |
|  | *Linanthus orcuttii* |  |  | AF027701 |
|  | *Linanthus parryae* |  |  | AF027699 |
|  | *Linanthus pusillus* | AF119439 | AF119465 |  |
|  | *Linanthus uncialis* |  |  | AF027696 |
|  | *Linanthus watsonii* |  |  | EU339818 |
|  | *Loeselia ciliata* |  |  | EU339759 |
|  | *Loeselia coerulea* |  |  | EU628275 |
|  | *Loeselia glandulosa* |  |  | EU628273 |
|  | *Loeselia involucrata* |  |  | EU339758 |
|  | *Loeselia pumila* |  |  | EU339756 |
|  | *Loeseliastrum depressum* |  |  | EU339762 |
|  | *Loeseliastrum matthewsii* |  |  | EU339763 |
|  | *Loeseliastrum schottii* |  |  | EU628277 |
|  | *Microgilia minutiflora* |  |  | EU339766 |
|  | *Microsteris gracilis* |  |  | EU339823 |
|  | *Navarretia atractyloides* |  |  | EF199708 |
|  | *Navarretia breweri* |  |  | EU339735 |
|  | *Navarretia capillaris* |  |  | EU339731 |
|  | *Navarretia divaricata* |  |  | U73871 |
|  | *Navarretia eriocephala* |  |  | U73874 |
|  | *Navarretia filicaulis* |  |  | U73862 |
|  | *Navarretia fossalis* |  |  | U73881 |
|  | *Navarretia hamata* |  |  | U73865 |
|  | *Navarretia heterandra* |  |  | U73875 |
|  | *Navarretia intertexta* |  |  | AF208223 |
|  | *Navarretia involucrata* |  |  | EU628293 |
|  | *Navarretia jaredii* |  |  | U73876 |
|  | *Navarretia jepsonii* |  |  | EU628291 |
|  | *Navarretia leptalea* |  |  | AY997928 |
|  | *Navarretia leucocephala* |  |  | U73887 |
|  | *Navarretia mellita* |  |  | EU339736 |
|  | *Navarretia myersii* |  |  | U73892 |
|  | *Navarretia nigelliformis* |  |  | U73878 |
|  | *Navarretia peninsularis* |  |  | U73872 |
|  | *Navarretia prolifera* |  |  | U73873 |
|  | *Navarretia prostrata* |  |  | U73893 |
|  | *Navarretia pubescens* |  |  | EU628292 |
|  | *Navarretia rosulata* |  |  | U73867 |
|  | *Navarretia saximontana* |  |  | U73896 |
|  | *Navarretia setiloba* |  |  | U73880 |
|  | *Navarretia sinistra* |  |  | AF208210 |
|  | *Navarretia squarrosa* |  |  | U73868 |
|  | *Navarretia subuligera* |  |  | U73894 |
|  | *Navarretia tagetina* |  |  | U73895 |
|  | *Navarretia viscidula* |  |  | U73869 |
|  | *Navarretia willamettensis* |  |  | U73897 |
|  | *Phlox alyssifolia* |  |  | AF167255 |
|  | *Phlox amoena* |  |  | AF167208 |
|  | *Phlox amplifolia* |  |  | AF167195 |
|  | *Phlox bifida bifida* |  |  | AF167175 |
|  | *Phlox bifida stellaria* |  |  | AF167176 |
|  | *Phlox buckleyi* |  |  | AF167180 |
|  | *Phlox carolina* |  |  | AF167183 |
|  | *Phlox cuspidata* |  |  | AF167251 |
|  | *Phlox diffusa* | AF119444 | AF119470 |  |
|  | *Phlox divaricata* |  |  | DQ006029 |
|  | *Phlox drummondii* |  |  | AF167252 |
|  | *Phlox floridana* |  |  | AF167218 |
|  | *Phlox glaberrima* |  |  | EU339825 |
|  | *Phlox hoodii* |  |  | AF167256 |
|  | *Phlox latifolia* |  |  | AF167192 |
|  | *Phlox longifolia* |  |  | AF167181 |
|  | *Phlox maculata* |  |  | AF167193 |
|  | *Phlox nana* |  |  | AF167202 |
|  | *Phlox nivalis* |  |  | AF167197 |
|  | *Phlox oklahomensis* |  |  | AF167199 |
|  | *Phlox paniculata* |  |  | AF167196 |
|  | *Phlox pattersonii* |  |  | AF167219 |
|  | *Phlox pilosa* |  |  | AF167220 |
|  | *Phlox pulcherrima* |  |  | AF167249 |
|  | *Phlox pulchra* |  |  | AF167194 |
|  | *Phlox roemeriana* |  |  | AF167203 |
|  | *Phlox stansburyi* |  |  | EU339824 |
|  | *Phlox stolonifera* |  |  | AF167179 |
|  | *Phlox subulata* |  |  | AF167177 |
|  | *Polemonium acutiflorum* |  |  | DQ320768 |
|  | *Polemonium boreale* |  |  | DQ320770 |
|  | *Polemonium brandegei* |  |  | DQ320771 |
|  | *Polemonium caeruleum* |  |  | EU339828 |
|  | *Polemonium californicum* |  |  | EU339826 |
|  | *Polemonium carneum* |  |  | DQ320777 |
|  | *Polemonium chartaceum* |  |  | DQ320780 |
|  | *Polemonium chinense* |  |  | DQ320781 |
|  | *Polemonium confertum* |  |  | DQ320782 |
|  | *Polemonium elegans* |  |  | DQ320783 |
|  | *Polemonium eximium* |  |  | DQ320790 |
|  | *Polemonium foliosissimum* |  |  | DQ320787 |
|  | *Polemonium grandiflorum* |  |  | DQ320788 |
|  | *Polemonium mexicanum* |  |  | DQ320789 |
|  | *Polemonium micranthum* |  |  | EU628254 |
|  | *Polemonium occidentale* |  |  | DQ320793 |
|  | *Polemonium pauciflorum* |  |  | EU339827 |
|  | *Polemonium pectinatum* |  |  | DQ320796 |
|  | *Polemonium pulcherrimum* |  |  | AF027704 |
|  | *Polemonium reptans* |  |  | DQ320805 |
|  | *Polemonium viscosum* |  |  | EU628256 |
|  | *Saltugilia australis* |  |  | EU628288 |
|  | *Saltugilia caruifolia* |  |  | EU339738 |
|  | *Saltugilia latimeri* |  |  | AY997945 |
|  | *Saltugilia splendens* |  |  | EU339737 |
|  | Outgroup: *Fouquieria splendens* |  |  | EU339721 |
| *Salvia* |  |  |  |  |
|  | *Salvia aegyptiaca* |  |  | DQ667285 |
|  | *Salvia aerea* |  |  | EU169469 |
|  | *Salvia aethiopis* |  |  | DQ667272 |
|  | *Salvia apiana* |  |  | DQ667214 |
|  | *Salvia arisanensis* |  |  | AB295100 |
|  | *Salvia aristata* |  |  | DQ667280 |
|  | *Salvia atrocyanea* |  |  | DQ667270 |
|  | *Salvia aucheri* |  |  | DQ667286 |
|  | *Salvia axillaris* |  |  | DQ667294 |
|  | *Salvia azurea* |  |  | DQ667317 |
|  | *Salvia bangii* |  |  | DQ667263 |
|  | *Salvia bowleyana* |  |  | EU592037 |
|  | *Salvia brevilabra* |  |  | EF373638. |
|  | *Salvia cabulica* |  |  | DQ667287 |
|  | *Salvia cacaliifolia* |  |  | DQ667259 |
|  | *Salvia californica* |  |  | DQ667213 |
|  | *Salvia campanulata* |  | FJ883500 |  |
|  | *Salvia canariensis* |  |  | DQ667256 |
|  | *Salvia candicans* |  |  | DQ667299 |
|  | *Salvia candidissima* |  |  | DQ667261 |
|  | *Salvia castanea* |  |  | EU169464 |
|  | *Salvia cavaleriei* |  |  | EF373620 |
|  | *Salvia cedrosensis* |  |  | DQ667228 |
|  | *Salvia chienii* |  |  | DQ132868 |
|  | *Salvia chinensis* |  |  | EF373647 |
|  | *Salvia chionopeplica* |  |  | DQ667227 |
|  | *Salvia clevelandii* |  |  | DQ667219 |
|  | *Salvia coccinea* |  |  | EU169484 |
|  | *Salvia cyclostegia* |  |  | EU169475 |
|  | *Salvia cynica* |  |  | DQ667332 |
|  | *Salvia dabieshanensis* |  | FJ883505 |  |
|  | *Salvia daghestanica* |  |  | DQ667258 |
|  | *Salvia davidsonii* |  |  | AF538919 |
|  | *Salvia deserta* |  |  | DQ132865 |
|  | *Salvia digitaloides* |  |  | EU169473 |
|  | *Salvia divinorum* |  |  | DQ667249 |
|  | *Salvia dolomitica* |  |  | DQ667322 |
|  | *Salvia dorrii* |  |  | DQ667229 |
|  | *Salvia dracocephaloides* |  |  | DQ667265 |
|  | *Salvia dugesii* |  | FJ883508 |  |
|  | *Salvia evansiana* |  |  | EF373623 |
|  | *Salvia farinacea* |  |  | EU169483 |
|  | *Salvia flava* |  |  | EF014349 |
|  | *Salvia fruticosa* |  | FJ883512 |  |
|  | *Salvia fulgens* |  |  | DQ667251 |
|  | *Salvia garipensis* |  |  | DQ667281 |
|  | *Salvia glabrescens* |  |  | AB295106 |
|  | *Salvia glutinosa* |  |  | DQ667250 |
|  | *Salvia graciliramulosa* |  |  | DQ667276 |
|  | *Salvia greatae* |  |  | DQ667215 |
|  | *Salvia haenkei* |  |  | DQ667271 |
|  | *Salvia hayatana* |  |  | AB295099 |
|  | *Salvia henryi* |  |  | DQ667216 |
|  | *Salvia hians* |  |  | DQ667239 |
|  | *Salvia honania* |  | FJ883513 |  |
|  | *Salvia hydrangea* |  |  | DQ667288 |
|  | *Salvia inconspicua* |  |  | DQ667298 |
|  | *Salvia isensis* |  |  | AB266241 |
|  | *Salvia japonica* |  |  | AB295096 |
|  | *Salvia kiangsiensis* |  | FJ883514 |  |
|  | *Salvia lasiantha* |  |  | DQ667300 |
|  | *Salvia lavanduloides* |  |  | DQ667297 |
|  | *Salvia leucophylla* |  |  | DQ667210 |
|  | *Salvia liguliloba* |  |  | EU592036 |
|  | *Salvia lutescens* |  |  | AB266243 |
|  | *Salvia maximowicziana* |  | FJ883516 |  |
|  | *Salvia meiliensis* |  | FJ883517 |  |
|  | *Salvia mellifera* |  |  | DQ667220 |
|  | *Salvia miltiorrhiza* |  |  | EU591978 |
|  | *Salvia mocinoi* |  |  | DQ667274 |
|  | *Salvia mohavensis* |  |  | DQ667212 |
|  | *Salvia munzii* |  |  | DQ667224 |
|  | *Salvia nipponica* |  |  | AB295103 |
|  | *Salvia officinalis* |  |  | DQ667225 |
|  | *Salvia omeiana* |  |  | EF373644 |
|  | *Salvia orbignaei* |  |  | DQ667279 |
|  | *Salvia ovalifolia* |  |  | DQ667315 |
|  | *Salvia oxyphora* |  |  | DQ667262 |
|  | *Salvia pachyphylla* |  |  | DQ667230 |
|  | *Salvia patens* |  |  | DQ667253 |
|  | *Salvia pauciflora* |  |  | EU169476 |
|  | *Salvia pentstemonoides* |  |  | DQ667221 |
|  | *Salvia personata* |  |  | DQ667269 |
|  | *Salvia platystoma* |  |  | DQ667277 |
|  | *Salvia plebeia* |  |  | AB295107 |
|  | *Salvia plectranthoides* |  |  | EU169479 |
|  | *Salvia polystachya* |  |  | DQ667292 |
|  | *Salvia pratensis* |  |  | EU169486 |
|  | *Salvia prionitis* |  | FJ883527 |  |
|  | *Salvia procurrens* |  |  | DQ667304 |
|  | *Salvia prunelloides* |  |  | DQ667275 |
|  | *Salvia przewalskii* |  |  | EF014346 |
|  | *Salvia pubescens* |  |  | DQ667296 |
|  | *Salvia pygmaea* |  |  | AB295098 |
|  | *Salvia ranzaniana* |  |  | AB287375 |
|  | *Salvia roborowskii* |  |  | EU169477 |
|  | *Salvia roemeriana* |  |  | DQ667211 |
|  | *Salvia rugosa* |  |  | DQ667290 |
|  | *Salvia rusbyi* |  |  | DQ667278 |
|  | *Salvia rypara* |  |  | DQ667266 |
|  | *Salvia sagittata* |  |  | DQ667260 |
|  | *Salvia sclarea* |  |  | DQ667222 |
|  | *Salvia scutellarioides* |  |  | DQ667327 |
|  | *Salvia semiatrata* |  |  | DQ667295 |
|  | *Salvia sessilifolia* |  |  | DQ667282 |
|  | *Salvia sonomensis* |  |  | DQ667218 |
|  | *Salvia sophrona* |  |  | DQ667268 |
|  | *Salvia splendens* |  |  | AF477788 |
|  | *Salvia stachydifolia* |  |  | DQ667267 |
|  | *Salvia substolonifera* |  |  | EF373646 |
|  | *Salvia summa* |  |  | DQ667217 |
|  | *Salvia sylvestris* |  |  | EU169485 |
|  | *Salvia taraxacifolia* |  |  | DQ667209 |
|  | *Salvia texana* |  |  | DQ667321 |
|  | *Salvia thymoides* |  |  | DQ667273 |
|  | *Salvia trichocalycina* |  |  | DQ667283 |
|  | *Salvia tricuspidata* |  |  | DQ667293 |
|  | *Salvia tricuspis* |  |  | EF373635 |
|  | *Salvia trijuga* |  |  | EF014347 |
|  | *Salvia umbratica* |  | FJ883532 |  |
|  | *Salvia vaseyi* |  |  | DQ667226 |
|  | *Salvia vasta* |  | FJ883533 |  |
|  | *Salvia verbascifolia* |  |  | DQ667264 |
|  | *Salvia whitehousei* |  |  | DQ667320 |
|  | *Salvia yunnanensis* |  | FJ546866 |  |
|  | Outgroup: *Melissa officianalis* |  |  | EU796895 |
| Saniculoideae |  |  |  |  |
|  | *Eryngium agavifolium* |  |  | EU168965 |
|  | *Eryngium aloifolium* |  |  | EU070601 |
|  | *Eryngium alpinum* |  |  | EU168966 |
|  | *Eryngium alternatum* |  |  | EU070603 |
|  | *Eryngium amethystinum* |  |  | EU168967 |
|  | *Eryngium aquifolium* |  |  | EU168968 |
|  | *Eryngium aromaticum* |  |  | EU070606 |
|  | *Eryngium articulatum* |  |  | EU070607 |
|  | *Eryngium balansae* |  |  | EU070608 |
|  | *Eryngium baldwinii* |  |  | EU168969 |
|  | *Eryngium beecheyanum* |  |  | EU168970 |
|  | *Eryngium billardierei* |  |  | EU168971 |
|  | *Eryngium bonplandii* |  |  | EU070609 |
|  | *Eryngium bourgatii* |  |  | EU168972 |
|  | *Eryngium brasiliense* |  |  | EU070611 |
|  | *Eryngium buchtienii* |  |  | EU070612 |
|  | *Eryngium bungei* |  |  | EU070613 |
|  | *Eryngium bupleuroides* |  |  | EU070614 |
|  | *Eryngium caeruleum* |  |  | EU168973 |
|  | *Eryngium caespitiferum* |  |  | EU070616 |
|  | *Eryngium campestre* |  |  | EU168974 |
|  | *Eryngium canaliculatum* |  | EU070618 |  |
|  | *Eryngium carlinae* |  |  | EU168975 |
|  | *Eryngium cervantesii* |  |  | AF031960 |
|  | *Eryngium chamissonis* |  |  | EU070623 |
|  | *Eryngium ciliatum* |  |  | EU070624 |
|  | *Eryngium coquimbanum* |  |  | EU070626 |
|  | *Eryngium corniculatum* |  |  | EU168976 |
|  | *Eryngium coronatum* |  |  | EU070629 |
|  | *Eryngium crassisquamosum* |  |  | EU070630 |
|  | *Eryngium creticum* |  |  | EU168977 |
|  | *Eryngium diffusum* |  |  | EU070632 |
|  | *Eryngium dilatatum* |  |  | EU168978 |
|  | *Eryngium divaricatum* |  |  | EU070633 |
|  | *Eryngium duriaei* |  | EU070634 |  |
|  | *Eryngium ebracteatum* |  |  | EU168979 |
|  | *Eryngium eburneum* |  |  | EU070637 |
|  | *Eryngium echinatum* |  |  | EU070638 |
|  | *Eryngium elegans* |  |  | EU070640 |
|  | *Eryngium eriophorum* |  |  | EU070641 |
|  | *Eryngium eurycephalum* | EU070642 |  |  |
|  | *Eryngium expansum* |  |  | EU168980 |
|  | *Eryngium falcifolium* |  |  | EU070643 |
|  | *Eryngium fernandezianum* |  |  | EU070644 |
|  | *Eryngium floribundum* |  |  | EU168981 |
|  | *Eryngium fluitans* |  |  | EU070646 |
|  | *Eryngium foetidum* |  |  | EU168982 |
|  | *Eryngium galioides* |  |  | EU168983 |
|  | *Eryngium ghiesbreghtii* |  |  | EU070649 |
|  | *Eryngium giganteum* |  |  | EU168984 |
|  | *Eryngium glaciale* |  |  | EU168985 |
|  | *Eryngium glomeratum* |  |  | EU168986 |
|  | *Eryngium glossophyllum* |  |  | EU070653 |
|  | *Eryngium goyazense* |  |  | EU070654 |
|  | *Eryngium gracile* |  |  | EU070655 |
|  | *Eryngium gramineum* |  |  | EU070656 |
|  | *Eryngium grosii* |  |  | EU168987 |
|  | *Eryngium heldreichii* |  |  | EU168988 |
|  | *Eryngium hemisphaericum* |  |  | EU070657 |
|  | *Eryngium hemsleyanum* |  |  | EU070658 |
|  | *Eryngium horridum* |  |  | EU168989 |
|  | *Eryngium humile* |  |  | EU168990 |
|  | *Eryngium huteri* |  |  | EU070621 |
|  | *Eryngium ilicifolium* |  |  | EU168991 |
|  | *Eryngium inaccessum* |  |  | EU070662 |
|  | *Eryngium incantatum* |  |  | EU070663 |
|  | *Eryngium integrifolium* |  |  | EU070664 |
|  | *Eryngium isauricum* |  |  | EU168992 |
|  | *Eryngium junceum* |  | EU070665 |  |
|  | *Eryngium juncifolium* |  |  | EU070666 |
|  | *Eryngium koehneanum* |  | EU070667 |  |
|  | *Eryngium kotschyi* |  |  | EU168993 |
|  | *Eryngium lacustre* |  |  | EU070668 |
|  | *Eryngium leavenworthii* |  |  | EU070669 |
|  | *Eryngium longifolium* |  |  | EU070670 |
|  | *Eryngium luzulaefolium* |  |  | EU070671 |
|  | *Eryngium macrocalyx* |  |  | EU070672 |
|  | *Eryngium madrense* |  |  | EU070673 |
|  | *Eryngium maritimum* |  |  | EU168994 |
|  | *Eryngium marocanum* |  |  | EU168995 |
|  | *Eryngium megapotamicum* |  |  | EU070677 |
|  | *Eryngium mesopotamicum* |  |  | EU070680 |
|  | *Eryngium mexiae* |  |  | EU070681 |
|  | *Eryngium mexicanum* |  |  | AF031961 |
|  | *Eryngium monocephalum* |  |  | EU070683 |
|  | *Eryngium montanum* | EU070684 |  |  |
|  | *Eryngium nasturtiifolium* |  |  | EU070685 |
|  | *Eryngium nudicaule* |  |  | EU070686 |
|  | *Eryngium ombrophilum* |  |  | EU070687 |
|  | *Eryngium ovinum* |  |  | EU168996 |
|  | *Eryngium palmatum* |  |  | EU070689 |
|  | *Eryngium palmeri* |  |  | EU070690 |
|  | *Eryngium pandanifolium* |  |  | EU168997 |
|  | *Eryngium paniculatum* |  |  | EU070694 |
|  | *Eryngium petiolatum* |  |  | EU168999 |
|  | *Eryngium pilularioides* |  |  | EU070695 |
|  | *Eryngium* cf. *pinnatifidum* |  |  | EU169000 |
|  | *Eryngium plantagineum* |  |  | EU169001 |
|  | *Eryngium planum* |  |  | EU169002 |
|  | *Eryngium pohlianum* |  |  | EU070697 |
|  | *Eryngium pristis* |  |  | EU169003 |
|  | *Eryngium prostratum* |  |  | EU070699 |
|  | *Eryngium proteiflorum* |  |  | EU070700 |
|  | *Eryngium pseudojunceum* |  |  | EU070701 |
|  | *Eryngium purpusii* |  |  | EU070702 |
|  | *Eryngium pyramidale* | EU070703 | EU070704 |  |
|  | *Eryngium rauhianum* |  |  | EU070706 |
|  | *Eryngium regnellii* |  |  | EU070709 |
|  | *Eryngium rojasii* |  |  | EU070710 |
|  | *Eryngium rostratum* |  |  | EU070711 |
|  | *Eryngium sanguisorba* |  |  | EU070713 |
|  | *Eryngium scaposum* |  |  | EU169004 |
|  | *Eryngium scirpinum* |  |  | EU169005 |
|  | *Eryngium sellowii* |  |  | EU070717 |
|  | *Eryngium serbicum* |  |  | EU070718 |
|  | *Eryngium serratum* |  |  | EU070719 |
|  | *Eryngium smithii* |  |  | EU070720 |
|  | *Eryngium sparganophyllum* |  |  | EU070721 |
|  | *Eryngium spiculosum* |  |  | EU070722 |
|  | *Eryngium spinalba* |  |  | EU169006 |
|  | *Eryngium subinerme* |  |  | EU070723 |
|  | *Eryngium tenue* |  |  | EU169007 |
|  | *Eryngium ternatum* |  |  | EU070726 |
|  | *Eryngium thorifolium* |  |  | EU169008 |
|  | *Eryngium tricuspidatum* |  |  | EU169009 |
|  | *Eryngium variifolium* |  |  | EU169010 |
|  | *Eryngium vaseyi* |  |  | EU070729 |
|  | *Eryngium venustum* |  |  | EU070731 |
|  | *Eryngium vesiculosum* |  |  | EU070732 |
|  | *Eryngium viviparum* |  |  | EU169011 |
|  | *Eryngium weberbaueri* | EU070734 |  |  |
|  | *Eryngium yuccifolium* |  |  | EU169012 |
|  | *Petagnaea gussonei* |  |  | EU070742 |
|  | *Sanicula arctopoides* |  |  | EU070743 |
|  | *Sanicula arguta* |  |  | AF031976 |
|  | *Sanicula bipinnata* |  |  | EU070744 |
|  | *Sanicula bipinnatafidia* |  |  | EU070745 |
|  | *Sanicula canadensis* |  |  | EU070746 |
|  | *Sanicula chinensis* |  |  | EU070747 |
|  | *Sanicula crassicaulis* |  |  | AJ012694 |
|  | *Sanicula deserticola* |  |  | AF031989 |
|  | *Sanicula elata* |  |  | AF031966 |
|  | *Sanicula epipactis* |  |  | EU169013 |
|  | *Sanicula europaea* |  |  | AF031964 |
|  | *Sanicula graveolens* |  |  | EU070748 |
|  | *Sanicula hoffmannii* |  |  | AF031995 |
|  | *Sanicula laciniata* |  |  | AF031998 |
|  | *Sanicula lamelligera* |  | AJ012695 |  |
|  | *Sanicula maritima* | AF032015 | AF032016 |  |
|  | *Sanicula mariversa* |  |  | AF031968 |
|  | *Sanicula moranii* |  |  | AF032012 |
|  | *Sanicula odorata* |  |  | EU070750 |
|  | *Sanicula orthacantha* |  |  | EU070751 |
|  | *Sanicula peckiana* |  |  | AF032001 |
|  | *Sanicula purpurea* |  |  | AF031971 |
|  | *Sanicula rubriflora* |  |  | AF077907 |
|  | *Sanicula sandwicensis* |  |  | AF031970 |
|  | *Sanicula saxatilis* |  |  | AF032005 |
|  | *Sanicula smallii* |  |  | EU070752 |
|  | *Sanicula tracyi* |  |  | AF032007 |
|  | *Sanicula tuberosa* |  |  | AF032010 |
|  | Outgroup: *Astrantia major* |  |  | AF031962 |
| *Sidalcea*, Etc. |  |  |  |  |
|  | *Calyculogygas uruguayensis* |  |  | AY591820 |
|  | *Eremalche exilis* |  |  | AY591823 |
|  | *Eremalche kernensis* |  |  | AJ849665 |
|  | *Eremalche parryi* |  |  | AJ849674 |
|  | *Eremalche rotundiflora* |  |  | AJ849677 |
|  | *Iliamna bakeri* |  |  | AF271169 |
|  | *Iliamna latibracteata* |  |  | AF271186 |
|  | *Malvastrum amblyphyllum* |  |  | AY591841 |
|  | *Malvastrum americanum* |  |  | AY591842 |
|  | *Malvastrum coromandelianum* | AJ274971 | AJ275002 |  |
|  | *Modiola caroliniana* |  |  | AY172190 |
|  | *Modiolastrum lateritium* |  |  | AY172191 |
|  | *Monteiroa glomerata* |  |  | AY591843 |
|  | *Sidalcea asprella* |  |  | AJ512146 |
|  | *Sidalcea calycosa* |  |  | AJ519996 |
|  | *Sidalcea campestris* |  |  | AJ520005 |
|  | *Sidalcea candida* |  |  | AJ304880 |
|  | *Sidalcea cusickii* |  |  | AJ520016 |
|  | *Sidalcea diploscypha* |  |  | AJ849680 |
|  | *Sidalcea glaucescens* |  |  | AJ520023 |
|  | *Sidalcea hartwegii* |  |  | AJ512150 |
|  | *Sidalcea hendersonii* |  |  | AJ304892 |
|  | *Sidalcea hickmanii* |  |  | AJ512151 |
|  | *Sidalcea hirsuta* |  |  | AJ512153 |
|  | *Sidalcea hirtipes* |  |  | AJ512154 |
|  | *Sidalcea keckii* |  |  | AJ849681 |
|  | *Sidalcea malachroides* |  |  | AJ512155 |
|  | *Sidalcea malviflora* |  |  | AJ512159 |
|  | *Sidalcea maxima* |  |  | AJ512163 |
|  | *Sidalcea multifida* |  |  | AJ304910 |
|  | *Sidalcea nelsoniana* |  |  | AF196545 |
|  | *Sidalcea neomexicana* |  |  | AJ520051 |
|  | *Sidalcea oregana* |  |  | AJ512166 |
|  | *Sidalcea pedata* |  |  | AJ304924 |
|  | *Sidalcea ranunculacea* |  |  | AJ520061 |
|  | *Sidalcea reptans* |  |  | AJ520071 |
|  | *Sidalcea robusta* |  |  | AJ304930 |
|  | *Sidalcea stipularis* |  |  | AJ304932 |
|  | *Sidalcea virgata* |  |  | AJ512169 |
|  | Outgroup: *Callirhoe involucrata* |  |  | AY591819 |
